# Non-invasive monitoring of arthritis treatment response via targeting of tyrosine-phosphorylated annexin A2 in chondrocytes

**DOI:** 10.1186/s13075-021-02643-3

**Published:** 2021-10-25

**Authors:** Shaw-Wei D. Tsen, Luke E. Springer, Krishna Sharmah Gautam, Rui Tang, Kexian Liang, Gail Sudlow, Amir Kucharski, Christine T. N. Pham, Samuel Achilefu

**Affiliations:** 1 Departments of Radiology, Washington University School of Medicine, St Louis, MO 63110 USA; 2 Division of Rheumatology, Washington University School of Medicine, St Louis, MO 63110 USA; 3 Departments of Biochemistry and Molecular Biophysics, Washington University School of Medicine, St Louis, MO 63110 USA; 4 Departments of Biomedical Engineering, Washington University School of Medicine, St Louis, MO 63110 USA

**Keywords:** Near-infrared fluorescent imaging, Treatment response monitoring, Inflammatory arthritis, Rheumatoid arthritis

## Abstract

**Background:**

The development and optimization of therapies for rheumatoid arthritis (RA) is currently hindered by a lack of methods for early non-invasive monitoring of treatment response. Annexin A2, an inflammation-associated protein whose presence and phosphorylation levels are upregulated in RA, represents a potential molecular target for tracking RA treatment response.

**Methods:**

LS301, a near-infrared dye-peptide conjugate that selectively targets tyrosine 23-phosphorylated annexin A2 (pANXA2), was evaluated for its utility in monitoring disease progression, remission, and early response to drug treatment in mouse models of RA by fluorescence imaging. The intraarticular distribution and localization of LS301 relative to pANXA2 was determined by histological and immunohistochemical methods.

**Results:**

In mouse models of spontaneous and serum transfer-induced inflammatory arthritis, intravenously administered LS301 showed selective accumulation in regions of joint pathology including paws, ankles, and knees with positive correlation between fluorescent signal and disease severity by clinical scoring. Whole-body near-infrared imaging with LS301 allowed tracking of spontaneous disease remission and the therapeutic response after dexamethasone treatment. Histological analysis showed preferential accumulation of LS301 within the chondrocytes and articular cartilage in arthritic mice, and colocalization was observed between LS301 and pANXA2 in the joint tissue.

**Conclusions:**

We demonstrate that fluorescence imaging with LS301 can be used to monitor the progression, remission, and early response to drug treatment in mouse models of RA. Given the ease of detecting LS301 with portable optical imaging devices, the agent may become a useful early treatment response reporter for arthritis diagnosis and drug evaluation.

**Supplementary Information:**

The online version contains supplementary material available at 10.1186/s13075-021-02643-3.

## Background

Rheumatoid arthritis (RA) is among the most common debilitating joint conditions in the United States, affecting up to 1% of the population [1–4]. In recent decades, therapeutic advances in disease-modifying antirheumatic drugs (DMARDs) have enabled inhibition of disease progression and made clinical remission an achievable goal [5–8]. However, a challenge remains in optimizing treatment regimens to reach such a state in the shortest duration of time, to minimize damage to the joints caused by elevated disease activity.

There is currently a lack of methods for monitoring early treatment response in RA patients, which has hampered accurate assessment of disease activity and posed a significant barrier to treatment adjustment [9]. In the context of RA, successful clinical management relies on proper selection of a therapy to which the patient will show response [10]. Unfortunately, the response to any particular drug is variable among patients, and there are no well-established methods to guide therapeutic choices [11]. The current clinical paradigm involves a series of trials and errors, where treatment response is monitored over a course of months relying on clinical scoring methods and imaging modalities such as X-rays and ultrasonography [10, 12, 13], which are insensitive to very early (i.e., <1 month) changes in disease activity. Positron emission tomography (PET), an experimental approach for arthritis imaging, provides greater sensitivity but has the disadvantage of increased radiation exposure [14]. Together, these limitations lead to additional costs and toxicities to the patient, and potentially worsens patient outcomes, since early treatment is ideal for optimal reduction in joint damage [15]. Importantly, the speed with which novel arthritis therapies can be evaluated in clinical trials depends on feedback regarding treatment efficacy. In these regards, there is a need for a non-invasive method that enables rapid assessment of therapeutic effect for RA.

Recently, fluorescence imaging (FI) has been explored as a novel method for diagnosis and tracking of arthritis treatment response in both preclinical models and humans [16–24]. The technique involves the administration of a near-infrared fluorophore, followed by the detection of accumulated fluorescence in affected joints. Near-infrared FI has several advantages over conventional imaging modalities including its low cost and the avoidance of ionizing radiation exposure. FI is well-suited for application to RA, where peripheral small joints in the extremities are involved. Agents that have been utilized in prior studies include non-targeted dyes (e.g. indocyanine green (ICG), Cy5.5) [18, 24]; dye-labeled monoclonal antibodies and small molecule ligands that bind macrophage or endothelial cell targets such as F4/80, E-selectin, αvβ3 integrin, and folate receptor s[25–28]; and enzyme-activatable probes. Non-targeted dyes such as ICG accumulate in inflamed joints primarily due to increased vascular permeability, resulting in low contrast in comparison to targeted agents [29, 30]. On the other hand, the existing targeted approaches which focus on activated macrophages and endothelial cells have reduced specificity as these cell types are not limited to RA.

We have developed a novel near-infrared dye-peptide conjugate termed LS301 (Fig. S1), which preferentially binds Tyr23-phosphorylated annexin A2 protein (pANXA2) with high affinity [31]. Annexin A2 is a calcium-dependent phospholipid-binding protein that plays a key role in inflammation by facilitating NF-κB activation and plasmin-dependent macrophage migration and infiltration in association with its binding partner S100A10 on the cell surface [32, 33]. Phosphorylation of annexin A2 at Tyr23 is a prerequisite for its translocation to the cell surface [32, 34, 35], where it is amenable to targeting by exogenously administered agents such as LS301. The expression and phosphorylation levels of annexin A2 are known to be upregulated in human RA patients relative to healthy controls, and overexpression of annexin A2 in joints promotes RA disease progression via induction of angiogenesis and joint destruction [36, 37]. Therefore, pANXA2 represents a potential target for molecular imaging of RA. In this report, we investigated the utility of pANXA2-targeted fluorescence imaging using LS301 for monitoring early treatment response in experimental inflammatory arthritis.

## Methods

### Chemicals and reagents

All the fluorenylmethyloxycarbonyl (Fmoc) amino acids and Fmoc-Lys (Boc)-Wang Resin were purchased from AAPPTec (Louisville, KY, USA). Dichloromethane (DCM), acetic acid, acetic anhydride, thioanisole, phenol, hydroxybenzotriazole (HOBt), N,N-diisopropylethylamine (DIEA), N-trityl-1,2-ethanediamine, phenol, thioanisol, dimethylformamide (DMF), N,N′-diisopropylcarbodiimide (DIC), trifluoroacetic acid (TFA), iodine, methyl tert-butyl ether (MTBE), O-(7-azabenzotriazol-1-yl)-N,N,N′,N′-tetramethyluronium hexafluorophosphate (HATU), and dexamethasone (DEX) were purchased from Sigma-Aldrich (St Louis, MO). Hematoxylin and eosin (H&E) stains were purchased from MilliporeSigma (St Louis, MO). Rabbit anti-pANXA2 (phospho-Tyr24) antibody was purchased from Signalway Antibody (College Park, MD). AlexaFluor 594-conjugated donkey anti-rabbit antibody was purchased from Thermo Fisher Scientific (Waltham, MA).

### Synthesis of LS301

LS301 (cypate-cyclic (^D^Cys-Gly-Arg-Asp-Ser-Pro-Cys)-Lys-OH) was synthesized as previously reported [31]. Briefly, the linear GRD peptide, H-^D^Cys (Acm)-Gly-Arg (Pbf)-Asp (tBu)-Ser (tBu)-Pro-Cys (Acm)-Lys (Boc)-OH, was prepared via a CEM Liberty Blue microwave peptide synthesizer (Matthews, NC, USA) on the Fmoc-Lys (Boc)-wang resin. The resin (0.1 mmol) was swelled in DCM for 1 h before use. Fmoc-amino acids (0.5 mmol, 5 eq), coupling reagent (HBTU, 0.5 mmol, 5 eq), and DIEA (1 mmol, 10 eq) were added to the resin and the mixture was reacted for 15 min under microwave irradiation (100W, 90°C). The resin was washed three times with DMF. Deprotection of Fmoc group was carried out by treatment of 20% piperidine/DMF for 5 min under microwave irradiation (100W, 90 °C). The peptidyl resin was washed and the peptide cyclized through the disulfide bridge with iodine (1.2 eq) in DMF for 90 min. Subsequently, cypate (3 eq) was conjugated to the cyclic peptide on solid support in the presence of DIC (5 eq) in DMF to afford the LS301 peptidyl resin. The resin was then treated with a cleavage cocktail of TFA: thioanisol: phenol: water (85:5:5:5, v/v/v/v) for 90 min at room temperature. The cleaved peptide product was concentrated *in vacuo* before purification by reverse-phase HPLC (Gilson, Middleton, WI, USA). Analytical HPLC was used to determine product purity (> 95%) and the compound identity was confirmed by electrospray ionization mass spectrometry on a Shimadzu LCMS-2020 Mass Spectrometer (Columbia, MD) with peaks observed at 1470 (M+1) and 735 (M+2/2).

### Animals

Male 5–7-week-old C57BL/6J mice were purchased from The Jackson Laboratory (Bar Harbor, ME) and housed in designated animal facilities. Mice were fed ad libitum and inspected regularly. All animal experiments were performed in compliance with guidelines and protocols approved by the Division of Comparative Medicine at Washington University in St. Louis. The animal protocol is subjected to annual review and approval by The Animal Studies Committee of Washington University.

### Arthritis mouse models

The K/BxN mice with spontaneous arthritis (F1) [38–40] were maintained in the laboratory of Dr. Christine Pham (Department of Internal Medicine, Washington University School of Medicine). To establish serum transfer arthritis (STA), male 6–8 weeks old C57BL/6J mice (The Jackson Lab, Bar Harbor, ME, USA) were injected intraperitoneally with 150–175 μL of serum derived from F1 mice (8–9 weeks old), with day 0 denoting the day of serum transfer/disease induction. Clinical manifestation of arthritis in each paw was assessed daily on a scale of 0—3 with 0 = no swelling or erythema, 1 = slight swelling or erythema, 2 = moderate erythema and swelling in multiple digits or entire paw, and 3 = pronounced erythema and swelling of an entire paw, with a maximum score of 12 per mouse as previously described [41]. Ankle thickness of two hind paws was measured using calipers. Animals were monitored for signs of distress during arthritis induction including their ability to move around the cage and access food/water.

### In vivo imaging

Animals were shaved and excess hair removed using commercially available hair removal cream. Mice were anesthetized with isoflurane for injection and imaging procedures. LS301 stock (or LS301-methotrexate or LS301-methylpresdnisolone conjugate stock) in dimethyl sulfoxide solution was diluted in phosphate-buffered saline to a final concentration of 60 μM and injected via tail vein into mice. In vivo near-infrared fluorescence using 785 nm excitation and 820 nm emission filters was assessed pre-injection, post-injection, and/or at indicated time points post-injection with a Pearl Small Animal Imaging System (LICOR Biotechnology, Lincoln, NE). Regions of interest (ROIs) for fluorescence quantitation were drawn and analyzed using the Pearl Small Animal Imaging System software.

### Treatment response monitoring studies

Experiments were performed in a blinded fashion, where the technician responsible for clinical assessment of paw score and ankle measurements was blinded to treatment groups. For studies on disease remission, mice with STA were injected with 6 nmol of intravenous LS301 on day 4 post-disease induction and imaged at 18h post LS301 injection using the Pearl Small Animal Imaging System as described above. Clinical paw scores and ankle measurements were obtained daily. On day 23 post-disease induction when the clinical paw scores of mice were near the baseline, mice were imaged again with 6 nmol intravenous LS301. Regions of interest (ROI) were quantitated, encompassing mouse upper extremities (all structures including and distal to the wrist) and lower extremities (all structures including and distal to the ankle), applied universally to all images using the Pearl software. Total extremity fluorescence (quantitated from ROIs) per mouse, averaged among *n* = 3 mice, was compared between groups. For studies on response to DEX treatment, mice with STA were injected with 6 nmol of intravenous LS301 on day 3 post disease induction and imaged at 18h post LS301 injection using the Pearl Small Animal Imaging System with *λ* = 785 nm (excitation)/820 nm (emission). Mice then received intraperitoneal DEX (10 mg/kg/dose) daily over a 6-day period. Clinical paw scores and ankle measurements were obtained daily. On day 9 (day of final DEX treatment), mice were imaged again with 6 nmol intravenous LS301. Regions of interest (ROI) were quantitated and analyzed as described above.

### Histological assessment

H&E staining, immunohistochemical staining for pANXA2, and microscopic analysis were performed as previously described [31]. Tissues of interest were harvested and frozen at -80°C in Optimal Cutting Temperature (OCT) media. Frozen sections were cut at 10 μm thickness, and slides were stored at – 40 °C. Consecutive sections were subjected to H&E and immunohistochemical analysis as follows. H&E staining was performed by the Musculoskeletal Histology and Morphometry Core, Washington University School of Medicine. Briefly, frozen sections were fixed for 10 min in 4% paraformaldehyde solution (Sigma, St. Louis, MO, USA) and stained with Harris hematoxylin for 90 s and with eosin (Sigma, St. Louis, MO) for 15 s, and then washed with tap water for 5 min. Some sections were stained with Safranin O and Fast Green counterstain (Musculoskeletal Histology and Morphometry Core, Washington University School of Medicine, St Louis, MO. For immunohistochemistry, slides were blocked with appropriate serum for 35 min or with 5% non-fat milk PBS (pH 7.4) overnight at 4 °C and incubated with primary antibody overnight at 4 °C or 1h at 37 °C. For pANXA2 studies, tissue sections were incubated with 1: 250 rabbit anti-pANXA2 (phospho-Tyr24) antibody (Signalway Antibody, College Park, MD). After washing twice with PBS, the tissue sections were incubated with 1:1000 AlexaFluor 594-conjugated donkey anti-rabbit antibody (Thermo Fisher Scientific, Waltham, MA) for 1 h at 25 °C respectively. Slides were washed again and stained with DAPI nuclear stain for 5 min (Thermo Fisher Scientific, Waltham, MA) for 45 min at 37 °C. After final washes, a coverslip with aqueous fluorescence-saving mounting media was applied prior to imaging. Slides were viewed using an Olympus B61 epifluorescence microscope (Olympus Corp., Tokyo, Japan) with filters/channels as follows: DAPI (Ex/Em = 330–385/420 nm), FITC (Ex/Em = 460–500/510–560 nm), Texas Red (Ex/Em = 542–582/604–644 nm), cypate (Ex/Em = 750–800/818–873 nm), using exposure times 1 to 30 s and sensitivity settings ISO200-ISO1600, with the same parameters used for control and treatment groups. ImageJ software (National Institutes of Health, Bethesda, MD, USA) was used for image processing.

### Immunoblotting

Tissues were homogenized using an ultrasonic processor in RIPA buffer (20mM Tris-HCl, pH 7.5, 150mM NaCl, 1mM Na_2_EDTA, 1mM EGTA, 1% NP-40, 1% sodium deoxycholate, 2.5mM sodium pyrophosphate, 1mM b-glycerophosphate, 1mM Na_3_VO_4_, 1 μg/ml leupeptin, 1mM PMSF). The tissue lysates were clarified by centrifugation. The protein was denatured in SDS gel-loading buffer (100mM Tris-HCl, 200mM DTT, 4% SDS, 0.2% bromophenol blue, and 20% glycerol) at 95 °C for 10 min and then separated on 12% SDS-polyacrylamide gels (50 μg of the tissue protein per sample). After electrophoresis, proteins were transferred to PVDF membrane using an EC140 Mini Blot Module (Thermo EC, Holbrook, NY) apparatus. The membrane was blocked for 1 h at room temperature in PBS containing 5% nonfat dry milk (w/v), 0.1% (v/v) Tween-20 (PBS-T), followed by incubation with Annexin A2 rabbit mAb (1:2000; Cat. 8235, Cell Signaling Tech.) or p-Annexin A2 mouse mAb (1:500; sc-135753, Lot# J2920; Santa Cruz) in PBS-T containing 3% nonfat dry milk (w/v) at 4°C overnight. After washing three times for 10 min each in PBS-T, the membrane was incubated for 1 h with diluted polyclonal goat anti-rabbit IgG or polyclonal goat anti-mouse IgG conjugated to horseradish peroxidase in PBS-T containing 3% nonfat dry milk (w/v). The membrane was then washed three times for 10 min each in PBS-T and developed using the chemiluminescence ECL kit (Pierce) according to the manufacturer’s instructions.

### Statistics

Differences between sample means were analyzed by two-tailed unpaired *t*-test (Microsoft Excel) with *p* < 0.05 as the threshold for statistical significance. Correlations between fluorescence measurements, clinical paw scores, and change in ankle thickness were analyzed using Pearson correlation (Microsoft Excel). For semiquantitative analysis of fluorescence, 3 mice per group allows 80% power to detect an effect size of 1.67 by 2-sided 2-sample *t*-test at alpha=5% [42].

## Results

### LS301 localizes to sites of joint inflammation in models of RA

**Fig. 1:**
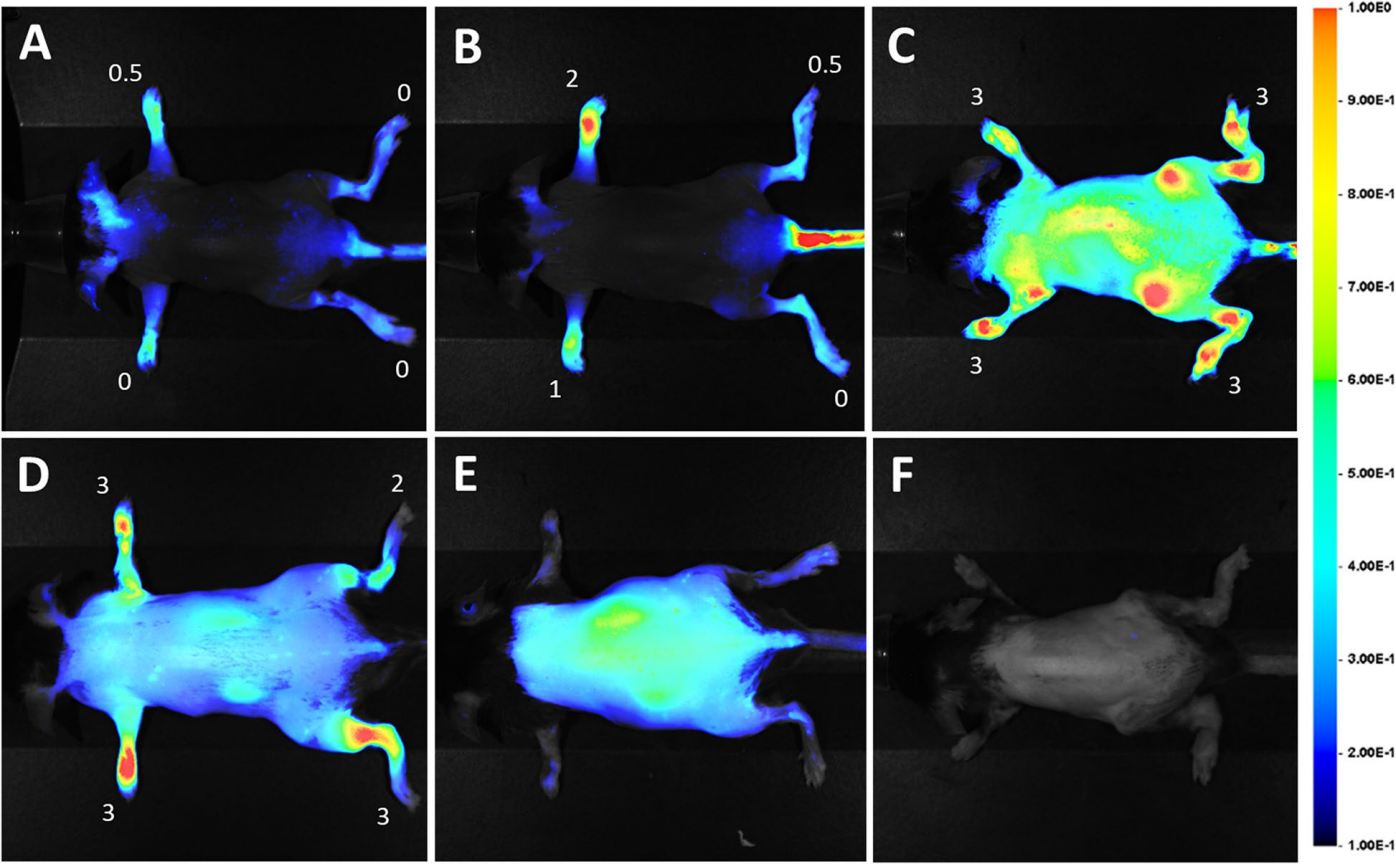
In vivo imaging of K/BxN spontaneous and serum transfer arthritis using LS301. Arthritic or control (non-diseased) mice were injected intravenously with 6 nmol LS301. Whole body near-infrared fluorescence images were taken on the Pearl animal imaging system with *λ* = 785 nm excitation and *λ* = 820 nm emission filters. Images shown are representative of at least two independent experiments and were taken at 18 h post-injection with mice in dorsal orientation. **A** Early-stage spontaneous K/BxN arthritis (3–4 weeks old F1 mice). **B** Intermediate stage spontaneous K/BxN arthritis (5–7 weeks old F1 mice). **C** Late-stage spontaneous K/BxN arthritis (9–10 weeks old F1 mice). **D** C57BL/6J mice with serum transfer arthritis (day 4 post disease induction). **E** Normal (control) C57BL/6J mice injected with LS301. **F** Intermediate stage spontaneous K/BxN arthritis (5–7 weeks old) F1 mice injected with cypate dye (control) in lieu of LS301. Numbers denote individual clinical paw scores at the time of imaging

**Fig. 2:**
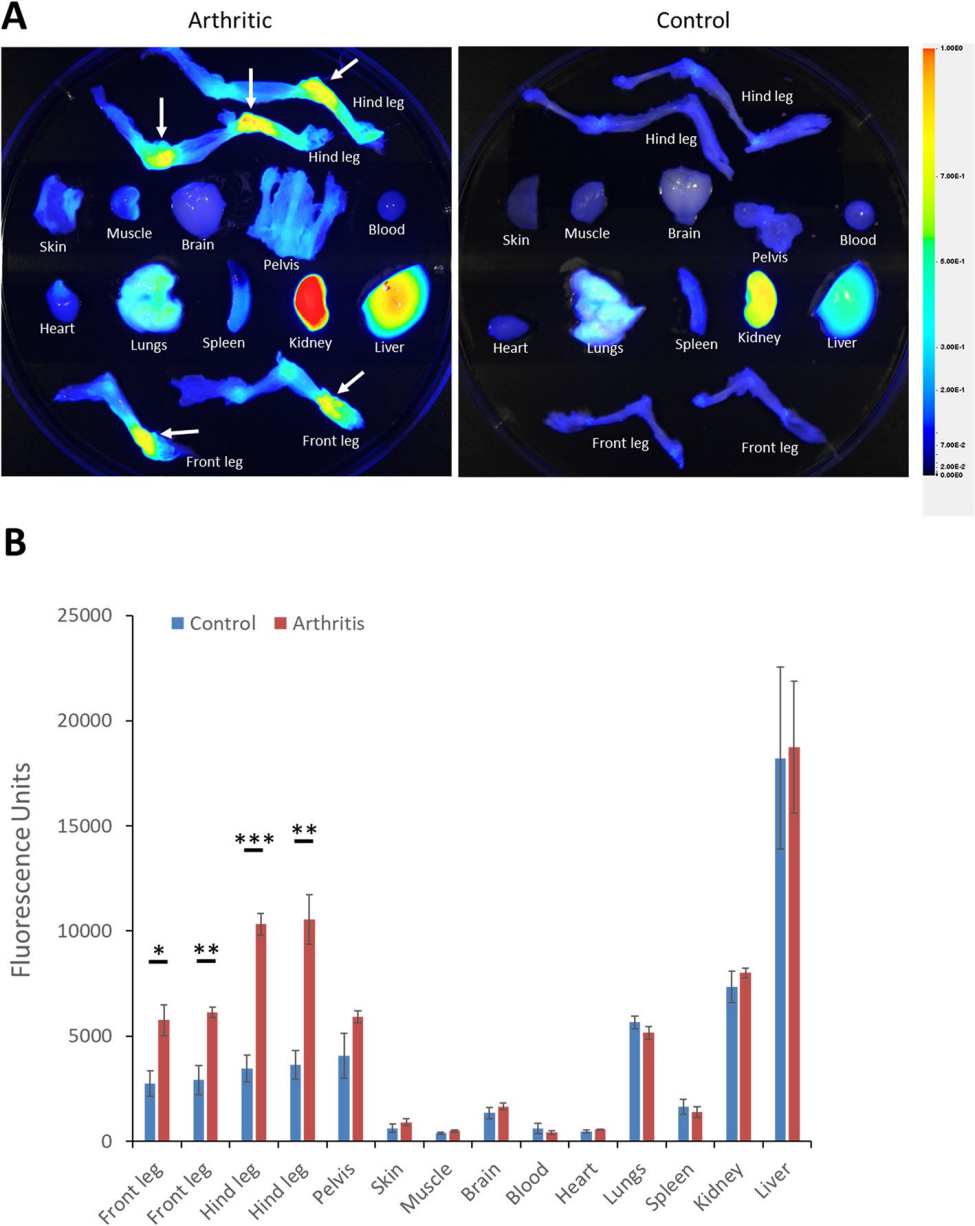
Typical LS301 biodistribution in arthritic mice. **A** Example ex vivo organ biodistribution of LS301 in C57BL/6J mice with serum transfer arthritis (left) or in control C57BL/6J mice (right) 18h after intravenous injection, as assessed by near-infrared fluorescence imaging on the Pearl animal imaging system. C57BL/6J mice with serum arthritis (days 5–6 post serum transfer) (left) or control C57BL/6J mice (right) were injected intravenously with 6 nmol LS301. Organs were harvested at 18h post injection. Near-infrared fluorescence images were taken on the Pearl animal imaging system with *λ* = 820 nm. Arrows denote detected areas of joint inflammation. **B** Quantification of fluorescence from individual organs from (**A**) (*n* = 4 mice per group). ROIs were drawn around each organ/limb of interest and quantitated using the Pearl animal imaging system software

In the K/BxN (F1) mice with spontaneous arthritis, inflammation occurs progressively in the paws, ankle, and knee joints leading to measurable local erythema and swelling (see Methods) [41]. F1 mice with severe arthritis (9–10 weeks old) were injected intravenously with LS301 and imaged 18h post-injection for near-infrared fluorescence using the Pearl Small Animal Imaging System. A time course assessment revealed the time point of optimal contrast at ~18 h post injection (Fig. S2), when LS301 was clearly seen to accumulate in regions of expected joint pathology in the extremities including paws and ankles. Using this time point, fluorescence imaging using LS301 in F1 mice with early (3–4 weeks old), intermediate (5–7 weeks old), or late-stage arthritis (9–10 weeks old) showed a positive correlation between fluorescence signal and disease severity as assessed by clinical paw scoring (Fig. 1A–C). Similar results were obtained in mice with K/BxN serum-transfer arthritis (STA) (Fig. 1D). Ex vivo biodistribution studies of LS301 in mice with induced arthritis confirmed selective accumulation of the agent in ankle and paw regions, with total fluorescence in individual limbs comparable to that of organs of excretion (liver, kidney) (Fig. 2). In contrast, healthy (non-diseased) control mice showed minimal LS301 accumulation in extremities (Figs. 1E and 2). To exclude increased blood flow/impeded circulation as the primary factor leading to compound accumulation in the diseased sites, we administered cypate dye alone (the dye component of LS301) to F1 arthritic mice (3–4 weeks old) and found no evidence of accumulation in extremities at 18 h (Fig. 1F).

### LS301 fluorescence correlates with arthritis disease severity in affected limbs

**Fig. 3:**
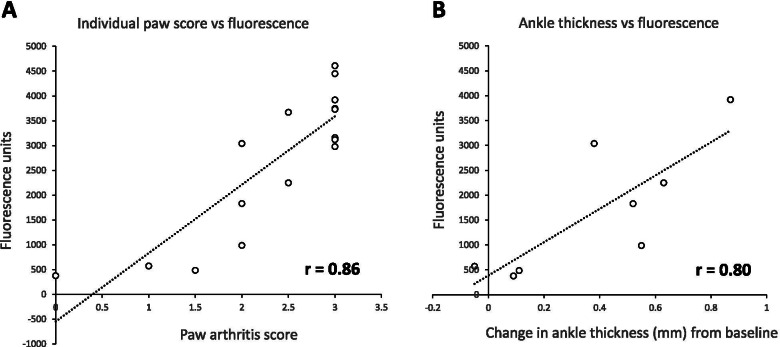
Correlation between LS301 fluorescent signal with disease severity in individual limbs. C57BL/6 mice (*n* = 4 per group) with serum transfer arthritis (day 4 post disease induction) were injected intravenously with 6 nmol LS301. Whole body near-infrared fluorescence images were taken on the Pearl animal imaging system with *λ* = 820 nm. Regions of interest (ROIs) were drawn to quantitate LS301 near-infrared fluorescence in each limb (paws and ankles). Individual limbs of the mice were scored for paw edema and measured for ankle thickness by calipers. Fluorescence was plotted against clinical scores (**A**) and ankle thickness measurements (**B**). Pearson’s correlation coefficient was calculated by the standard equation using Microsoft Excel software

We next assessed the utility of LS301 as an imaging modality for monitoring disease activity. In a cohort of mice with STA, total fluorescence in each affected limb area encompassing the animal’s upper extremities (all structures including and distal to the wrist) or lower extremities (all structures including and distal to the ankle), as determined using the Pearl software via ROI quantification (Fig. S3), was plotted against clinical paw scores and ankle thickness measurements. As described in Methods, experiments were performed in blinded fashion where the technician responsible for clinical assessment of paw score and ankle measurements was blinded to treatment groups. We found a significant positive correlation between LS301 fluorescence and disease severity by both parameters (r=0.86 and r=0.80 respectively) (Fig. 3). In addition, LS301 fluorescence signal successfully discriminated between diseased and healthy (non-diseased) extremities using a threshold total clinical paw score of 1. These results demonstrate the potential of LS301 as a useful tool for monitoring the severity of disease activity and progression.

### LS301 tracks disease progression and regression

**Fig. 4:**
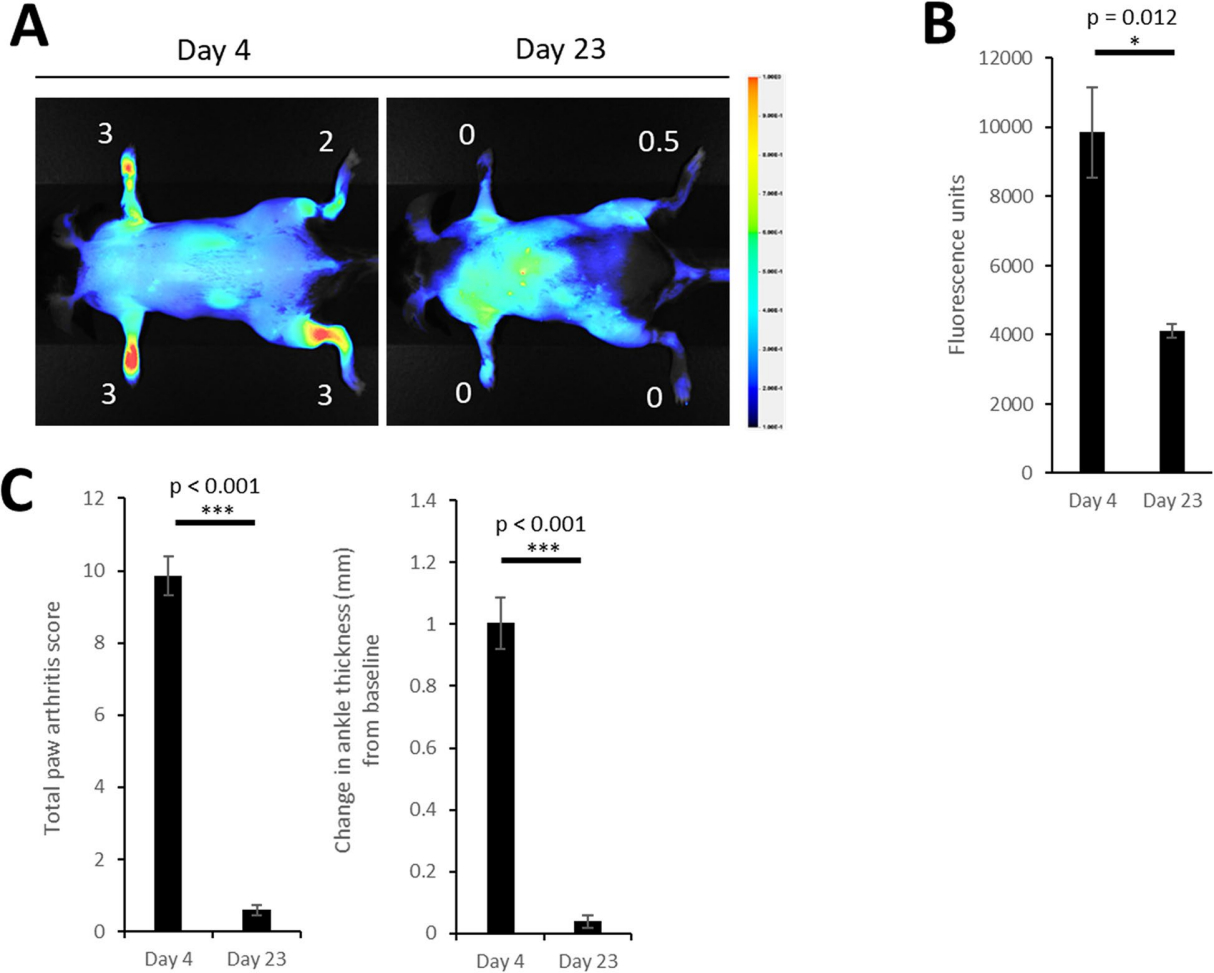
Monitoring arthritis disease remission using LS301. **A** Representative fluorescence images of arthritic mice imaged with LS301 at disease day 4 (left) and after disease remission (right). C57BL/6 mice (*n* = 3 per group) with induced arthritis (STA) were imaged with 6 nmol intravenous LS301 at day 4 post disease induction. At day 23 post-disease induction when the clinical paw scores of mice were near baseline, mice were imaged again with 6 nmol intravenous LS301. Whole body near-infrared fluorescence images were taken on the Pearl animal imaging system with *λ* = 820 nm. Images were taken at 18h post-injection with mice in dorsal orientation and depict representative independent replicates. Numbers denote individual clinical paw scores at the time of imaging. **B** Comparison of average total extremity fluorescence (quantitated from ROIs) per mouse described in **A**. **C** Total paw arthritis scores and change in ankle thickness from baseline of mice described in **A**

Current paradigms in RA management generally require trial periods on a time scale of months before the therapeutic response of a patient to DMARDs can be determined via clinical scoring, imaging, and/or inflammatory markers. This delay leads to increased risks of disease progression during this interval as well as unnecessary drug toxicities and financial expenses. Therefore, an imaging technique capable of reporting early response to drug treatment would have a significant translational impact. As a prelude to further studies, we confirmed that LS301 administration alone at the imaging dose (6 nmol) did not significantly affect disease progression (Fig. S4). First, to assess the use of LS301 in monitoring disease activity, we imaged mice with STA near the peak of their disease (day 4 post serum transfer) using LS301. At day 23, when clinical scores had returned to their baseline (pre-disease induction) levels, the mice were imaged again using LS301. As shown in Fig. 4, LS301 fluorescence correlated with disease progression and regression in these mice as orthogonally confirmed by clinical paw scoring.

**Fig. 5:**
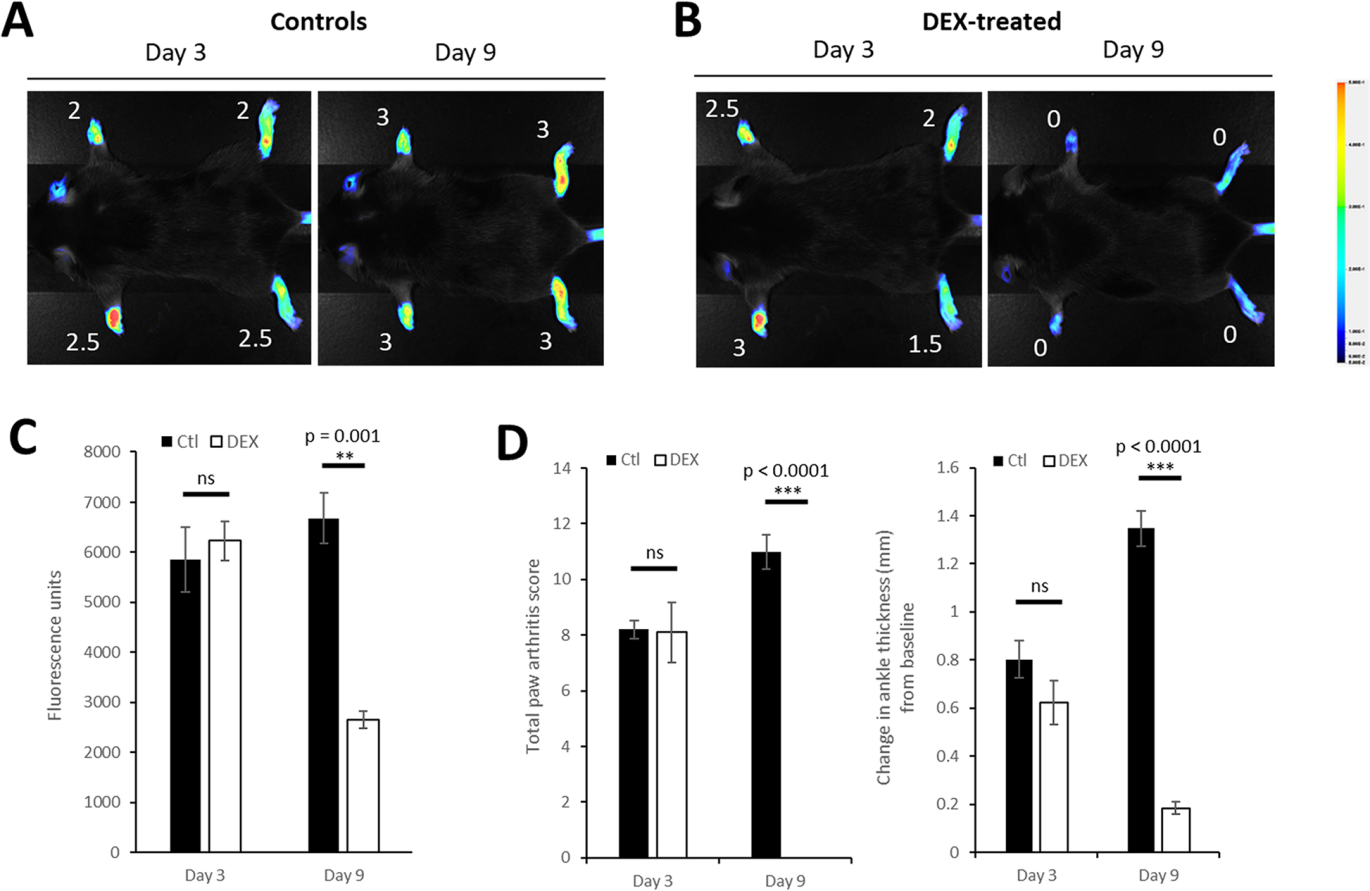
Monitoring DEX-associated treatment response using LS301. Representative fluorescence images of arthritic mice imaged with LS301 before (left) and after (right) dexamethasone (DEX) treatment. C57BL/6 mice with induced arthritis (STA) (*n* = 3 per group) were imaged with 6 nmol intravenous LS301 at day 3 post-disease induction. Mice then **A** remained untreated as controls or **B** were treated with daily doses of intraperitoneal dexamethasone (10 mg/kg/dose) over a 6-day period. At day 9 post-disease induction, mice were imaged again with 6 nmol intravenous LS301. Whole body near-infrared fluorescence images were taken on the Pearl animal imaging system with *λ* = 820 nm. Images were taken at 18h post-injection with mice in dorsal orientation. Numbers denote individual clinical paw scores at the time of imaging. **C** Comparison of average total extremity fluorescence (quantitated from ROIs) per mouse described in **A** and **B**. **C** Total paw arthritis scores and change in ankle thickness from baseline of mice described in **A** and **B**

Next, to evaluate the use of LS301 in monitoring early treatment response, we imaged mice with STA (day 3 post disease induction) using LS301. Mice then received either no treatment (control) or six daily treatments of dexamethasone (DEX). After the conclusion of the treatment cycle (day 9 post disease induction), mice were imaged again with LS301. While control mice continued to show disease progression as assessed by a high level of LS301 fluorescence and clinical paw scores (Fig. 5A, C–D), mice treated with DEX showed reductions in disease severity that correlated closely with LS301 fluorescence (Fig. 5B, C–D). Taken together, these results demonstrate the utility of LS301 fluorescence for monitoring early treatment response for RA.

### LS301 accumulates within chondrocytes and articular cartilage in arthritic mice

**Fig. 6:**
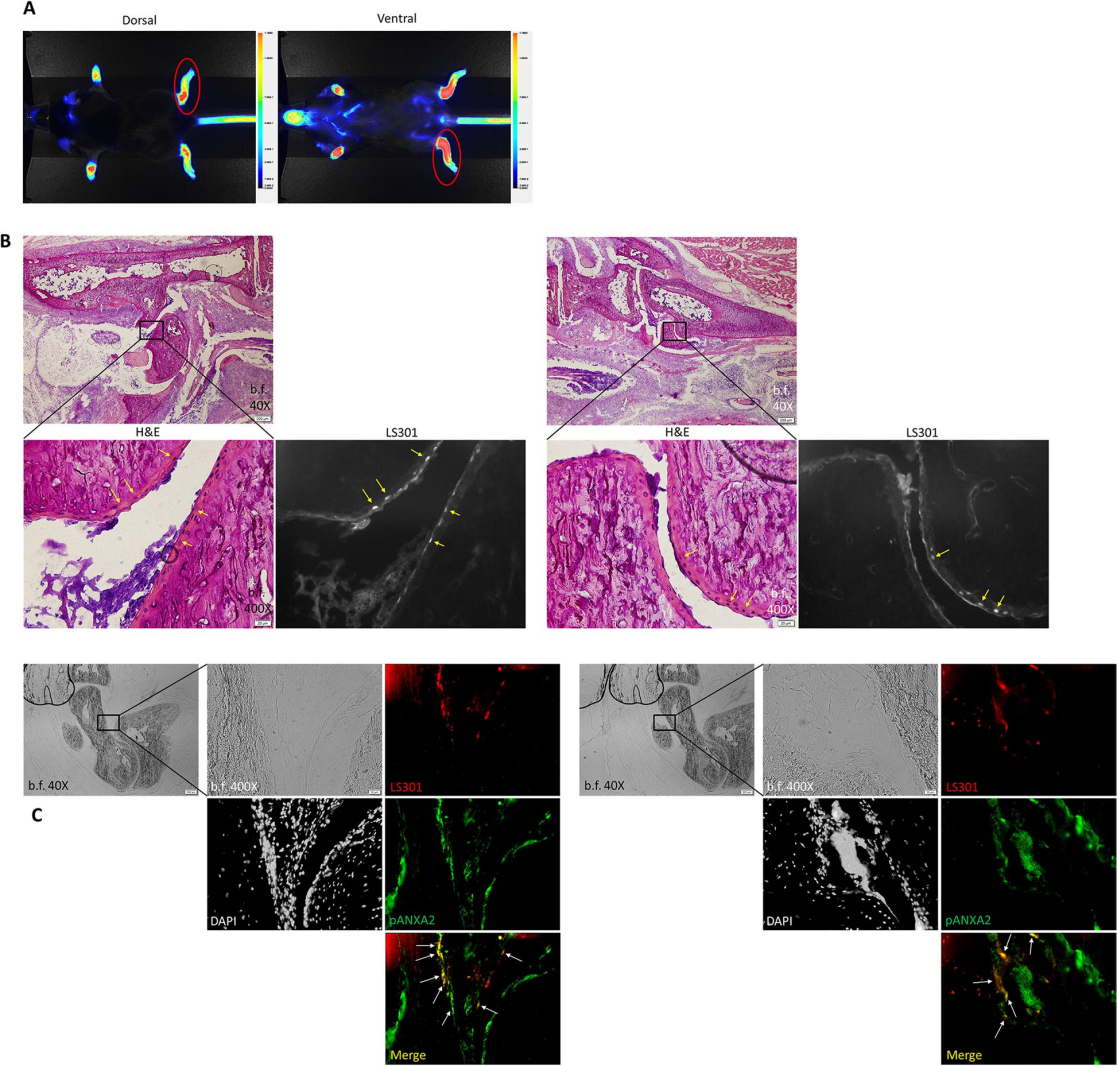
Cellular localization of LS301 in the arthritic mouse paws. C57BL/6 mice with serum transfer arthritis were intravenously injected with 6 nmol LS301 at day 4 post disease induction. 6 h after LS301 injection, whole body near-infrared fluorescence images were takePlease check if the figure captions are presented correctly.n on the Pearl animal imaging system with *λ* = 820 nm, and subsequently paws and ankles were harvested and frozen for sectioning. Sections were stained with H&E, or left unstained and examined for LS301 fluorescence by microscopy under the cypate channel (Ex/Em 775±25nm/845±28nm) (red) and/or viewed for pANXA2 (AlexaFluor 594 fluorescence) (green) under the Texas Red channel (Ex/Em 562±20nm/624±20nm). Images are representative of results from at least two independent experiments. **A** Example Pearl near-infrared fluorescence images of mice immediately prior to limb harvest. Red circles denote representative examples of the limb area harvested for sectioning. **B** H&E and fluorescence images from corresponding regions of articular cartilage in mouse ankle. Arrows indicate example regions of LS301 accumulation. **C** Fluorescence microscopy images from corresponding regions of articular cartilage in mouse ankle. Sections were stained with DAPI and anti-pANXA2 Ab/AlexaFluor 594-conjugated secondary Ab. Shown are DAPI (grayscale), LS301 fluorescence (red), and pANXA2 (green). Arrows denote LS301-pANXA2 colocalization

To assess the tissue distribution of LS301, we administered LS301 to mice with STA and harvested mouse ankle tissues for fluorescent and immunohistochemical (IHC) analyses (Fig. 6A). H&E-stained sections of ankles showed clear localization of LS301 fluorescence within chondrocytes and articular cartilage by superimposition of fluorescence images with H&E-stained images (Fig. 6B). In contrast, minimal or no LS301 signal above background was detected in other tissue regions including skin, connective tissue, muscle, bone, or bone marrow (Figs. S5 and S6). Previously, we found that LS301 binds with high affinity to pANXA2, which is known to be upregulated in arthritic cartilage [36, 37, 43]. IHC staining of ankle sections for pANXA2 revealed significant colocalization of LS301 fluorescence with pANXA2 expression within articular cartilage (Fig. 6C).

**Fig. 7:**
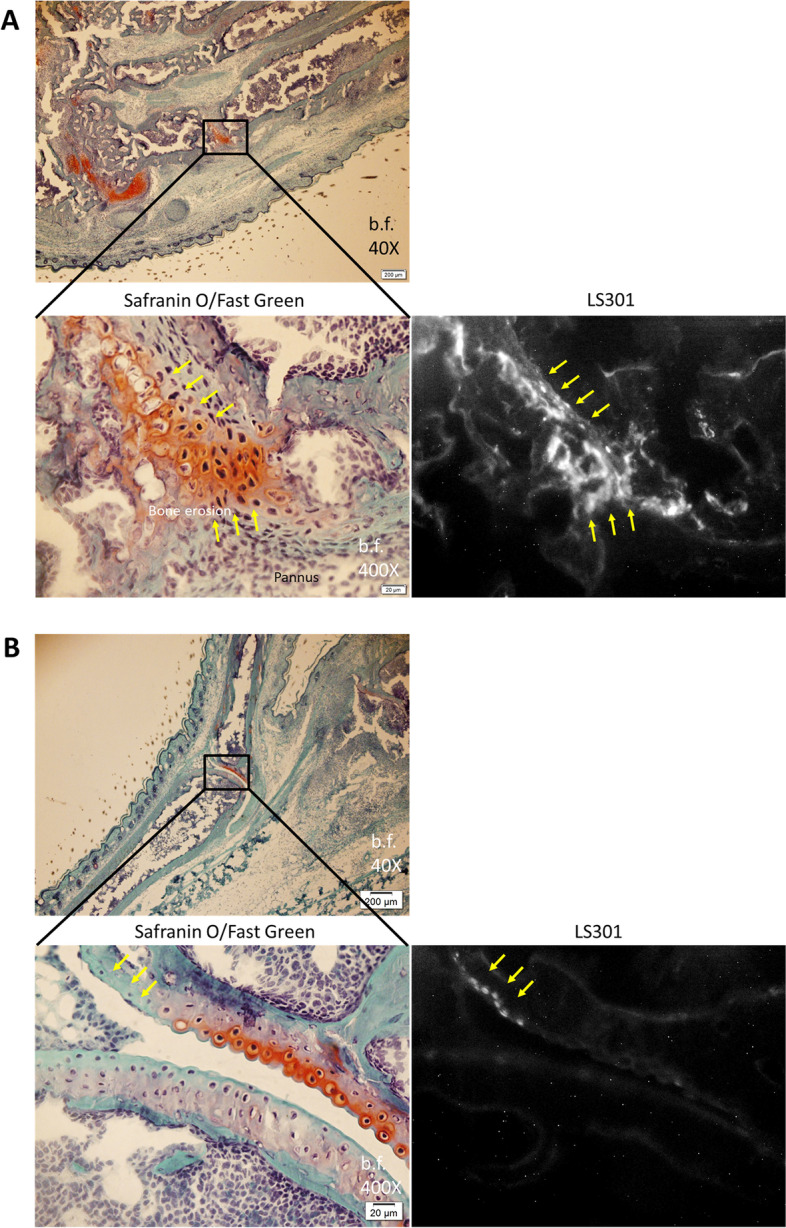
LS301 association with cartilage damage. K/BxN F1 mice (6–7 weeks old) (*n* = 2 mice per group) were injected intravenously with 6 nmol LS301. Six hours after LS301 injection, paws and ankles were harvested and frozen for sectioning. Sections were stained with Safranin O/Fast Green and viewed for LS301 fluorescence by microscopy under the cypate channel (Ex/Em 775±25nm/845±28nm). **A** Images from regions associated with pannus/bone erosion. Arrows indicate areas of LS301 accumulation. **B** Images showing preferential association of LS301 with regions of cartilage damage (loss of Safranin O staining). Arrows indicate areas of LS301 accumulation

To further evaluate whether there is a correlation between cartilage damage and LS301 accumulation in the joint, we examined paw sections stained with Safranin O. We found that LS301 localizes to areas of pannus/bone erosion (Fig. 7A). Moreover, LS301 preferentially localizes to regions of damaged cartilage, as evidenced by loss of Safranin O staining (Fig. 7B). These results indicate that LS301 targets chondrocytes in areas of damaged cartilage.

## Discussion

In lieu of the current trial-and-error practice for choosing RA therapeutics, a system for rapid assessment of arthritis treatment efficacy would enable proper drug selection at the crucial early stages when the disease course is most amenable to drug treatment. Such a system would also provide a much-needed platform for rapid screening and development of novel therapeutics. We describe here a novel near-infrared molecular imaging agent termed LS301, which was previously shown to target pANXA 2[31], and demonstrate its utility in monitoring RA disease activity, progression, and early (6 days) molecular response to treatment in the context of fluorescence imaging. We have shown in mouse models of RA that the therapeutic response to drug treatment can be tracked on a time span of several days using LS301, which if successfully translated to clinical application would represent a substantial improvement over the months required by the current scoring and imaging methods. Near-infrared FI does not involve ionizing radiation, and thus benefits from increased safety relative to imaging methods such as PET, conventional radiography, and CT. A further translational advantage of LS301 compared to other targeted fluorescent agents is that this agent has advanced beyond preclinical testing and is currently undergoing a clinical trial for cancer imaging (# NCT02807597) at the time of this report.

Of note, we observed that LS301 localizes preferentially in chondrocytes within the arthritic articular cartilage (Fig. 6b). As the sole producers of cartilaginous matrix and as mediators of inflammation, chondrocytes represent an important future cellular target for therapy in RA as well as osteoarthritis [44–46]. Chondrocytes have been a relatively overlooked target in fluorescence imaging approaches for RA, which have typically focused on inflammatory cells such as macrophages [27, 28]. Thus far, efforts to deliver drugs to chondrocytes have had limited success due to rapid clearance of molecules from the joint space following injections, the avascular nature of cartilage tissue, and the location of chondrocytes in the relatively inaccessible middle and deep zones of cartilage [47, 48]. The observed penetration of LS301 into chondrocytes raises the possibility of specific drug delivery to these cells via conjugation with LS301. Further studies are warranted to elucidate the unique mechanism(s) enabling LS301 to traverse the biological barriers posed by the cartilaginous tissues.

In our biodistribution studies, we note that there exists some degree of natural inter-individual variability in organ LS301 uptake. For example, in a proportion of the mice, a possible nonsignificant trend toward increased kidney signal in arthritic mice when compared to controls was observed. It was previously reported that G6PI-antibody immune complexes, which are a key component in the pathogenesis of our serum transfer arthritis model, also localize to the kidney glomeruli [49]. It is possible that in some individuals, such a process could result in inflammation/elevated pANXA2 expression and some degree of LS301 accumulation in this region.

In line with a shift toward precision medicine, strategies for targeting drugs to sites of inflammation could enhance the potential of existing rheumatologic drugs by increasing local delivery and reducing off-target toxicity. Although intra-articular injection of therapeutics can achieve high local concentrations, this approach becomes impractical in cases where multiple joints are involved such as in RA. Our observation that LS301 accumulates in target areas of inflammation following administration suggests its potential to circumvent these challenges. The unique chemical structure of LS301 (Fig. S1) enables it to readily serve as a covalent drug carrier via linkage with small molecule drugs or peptides. Further development of LS301 and its conjugates may enable the development of novel first-in-class theranostic agents not only for RA but also for OA and other arthritides.

## Conclusions

We demonstrate that FI using the pANXA2-targeting agent LS301 can be used to monitor the progression, remission, and early response to drug treatment in mouse models of RA. The observed selectivity of LS301 for arthritic lesions and the association of LS301 with chondrocytes in vivo provides a novel potential avenue for molecularly targeted imaging and drug evaluation.

## Supplementary Information

**Additional file 1: Figure S1**. Chemical structure of LS301.

**Additional file 2: Figure S2**. LS301 time course imaging in arthritic mice. Mice with spontaneous K/BxN arthritis (9-10 weeks old) (n=1 per group) were injected intravenously with 6 nmol LS301. Whole body near-infrared fluorescence images were taken at the indicated times on the Pearl animal imaging system with λ= 820 nm.

**Additional file 3: Figure S3**. Regions of interest (ROIs) used for quantitation of fluorescence in individual arthritic limbs. Representative example of regions of interest (ROI) encompassing mouse upper extremities (all structures distal to and including the wrist) and lower extremities (all structures distal to and including the ankle) that were quantitated for LS301 fluorescence using the Pearl animal imaging system software.

**Additional file 4: Figure S4**. Effect of imaging-dose (6 nmol) LS301 on arthritic disease progression. C57BL/6 mice with serum transfer arthritis (n=5 per group) were treated daily with 6 nmol intravenous LS301 from days 0 through 4 post disease induction, with daily 4h post-injection imaging. Whole body near-infrared fluorescence images were taken on Pearl Imaging System with λ= 820 nm. Aggregate clinical paw scores were determined daily. Arrows denote the timing of LS301 treatments.

**Additional file 5: Figure S5**. Controls for immunohistochemical staining. C57BL/6 mice (n=1) with serum induced arthritis were injected intravenously with 6 nmol LS301 at day 4 post disease induction. 6h after LS301 injection, whole body near-infrared fluorescence images were taken on the Pearl animal imaging system with λ= 820 nm, and subsequently paws and ankles were harvested and frozen for sectioning. Sections were incubated with AlexaFluor 594-conjugated secondary antibody only and viewed for fluorescence by microscopy under the Texas Red channel (Ex/Em 562±20nm/624±20nm).

**Additional file 6: Figure S6**. Tissue distribution of LS301 in extraarticular regions of the mouse extremity. C57BL/6 mice with serum transfer arthritis were injected intravenously with 6 nmol LS301 at day 4 post disease induction. 6h after LS301 injection, paws and ankles were harvested and frozen for sectioning. Sections were stained with H&E and viewed for LS301 fluorescence by microscopy under the cypate channel (Ex/Em 775±25nm/845±28nm) (red). Shown are representative H&E and fluorescence images from the indicated tissues in mouse ankle. Images are representative of at least two independent experiments. (A) Skin/dermis. (B) Connective tissue. (C) Muscle. (D) Bone. (E) Bone marrow.

**Additional file 7: Figure S7**. Relative expression levels of pANXA2 and ANXA2 in ankle and foot tissues of arthritic mice. C57BL/6 mice with serum transfer arthritis (n=1 per group) were sacrificed at day 8 post disease induction and ankle and foot tissue were harvested, homogenized and subjected to immunoblotting analysis for pANXA2 and ANXA2. Left: Immunoblot data for pANXA2 and ANXA2 in arthritic vs. control mouse tissue. Right: Corresponding signal quantitation using ImageJ software.

**Additional file 8: Figure S8**. Biodistribution of LS301 shown on different scales. Shown is an example LS301 organ biodistribution from C57BL/6 mice with STA or control mice injected intravenously with LS301 (n=4 per group), shown at two different scales. Fluorescence images shown were acquired using the Pearl Small Animal Imager. Left: Arthritic mouse LS301 organ biodistribution; right: control mouse LS301 organ biodistribution.

**Additional file 9: Figure S9**. Representative image depicting organ ROI delineation on the Pearl Small Animal Imager software. Shown is an example LS301 organ biodistribution from C57BL/6 mice with STA injected intravenously with LS301 (n=4). ROIs were drawn using the freehand shape tool on the Pearl Small Animal Imager.

**Additional file 10: Figure S10**. Raw data from ROI quantitation (Fig. 2B).

## Data Availability

The datasets used and/or analyzed during the current study are available from the corresponding author on reasonable request.

## Abbreviations

RA: Rheumatoid arthritis
DMARDs: Disease-modifying antirheumatic drugs
FI: Fluorescence imaging
ICG: Indocyanine green
pANXA2: Tyr23-phosphorylated annexin A2
STA: Serum transfer arthritis
