# Supplementary figures and images for: Non-invasive monitoring of arthritis treatment response via targeting of tyrosine-phosphorylated annexin A2 in chondrocytes
Source: Arthritis Res Ther. 2021 Oct 25;23:265. doi: 10.1186/s13075-021-02643-3 (PMC8543875; doi:10.1186/s13075-021-02643-3)

Figure S1 | Chemical structure of LS301.

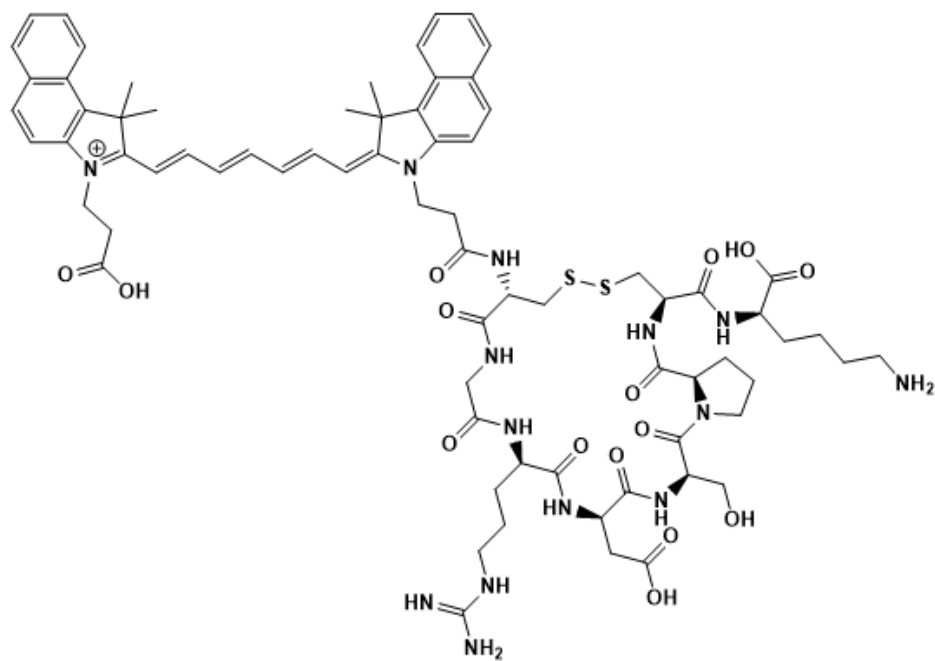

Supplement: Supplementary file 1 — Additional file 1: Figure S1. Chemical structure of LS301. [file 13075_2021_2643_MOESM1_ESM.pdf]

**Figure S2 | LS301 time course imaging in arthritic mice.**

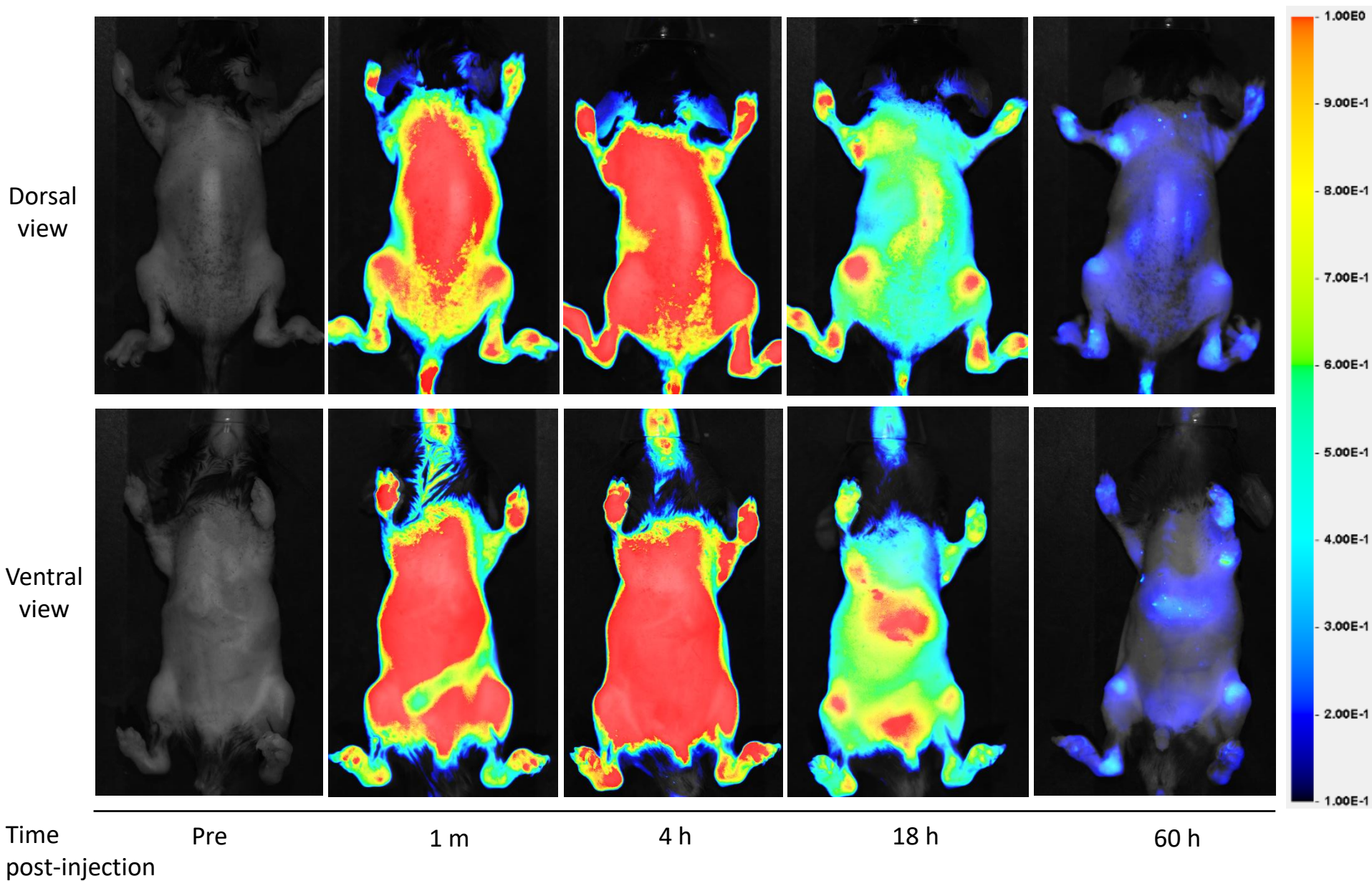

Supplement: Supplementary file 2 — Additional file 2: Figure S2. LS301 time course imaging in arthritic mice. Mice with spontaneous K/BxN arthritis (9-10 weeks old) (n=1 per group) were injected intravenously with 6 nmol LS301. Whole body near-infrared fluorescence images were taken at the indicated times on the Pearl animal imaging system with λ= 820 nm. [file 13075_2021_2643_MOESM2_ESM.pdf]

**Figure S4 | Effect of imaging-dose (6 nmol) LS301 on arthritic disease progression.**

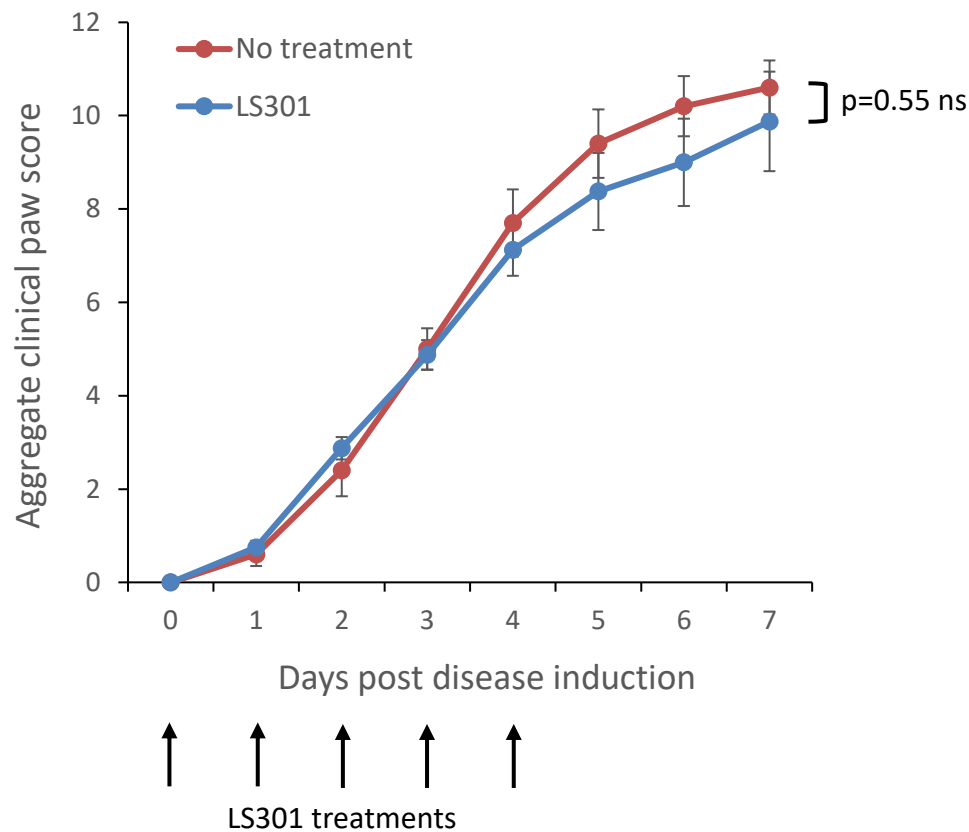

Supplement: Supplementary file 4 — Additional file 4: Figure S4. Effect of imaging-dose (6 nmol) LS301 on arthritic disease progression. C57BL/6 mice with serum transfer arthritis (n=5 per group) were treated daily with 6 nmol intravenous LS301 from days 0 through 4 post disease induction, with daily 4h post-injection imaging. Whole body near-infrared fluorescence images were taken on Pearl Imaging System with λ= 820 nm. Aggregate clinical paw scores were determined daily. Arrows denote the timing of LS301 treatments. [file 13075_2021_2643_MOESM4_ESM.pdf]

**Figure S5 | Controls for immunohistochemical staining.**

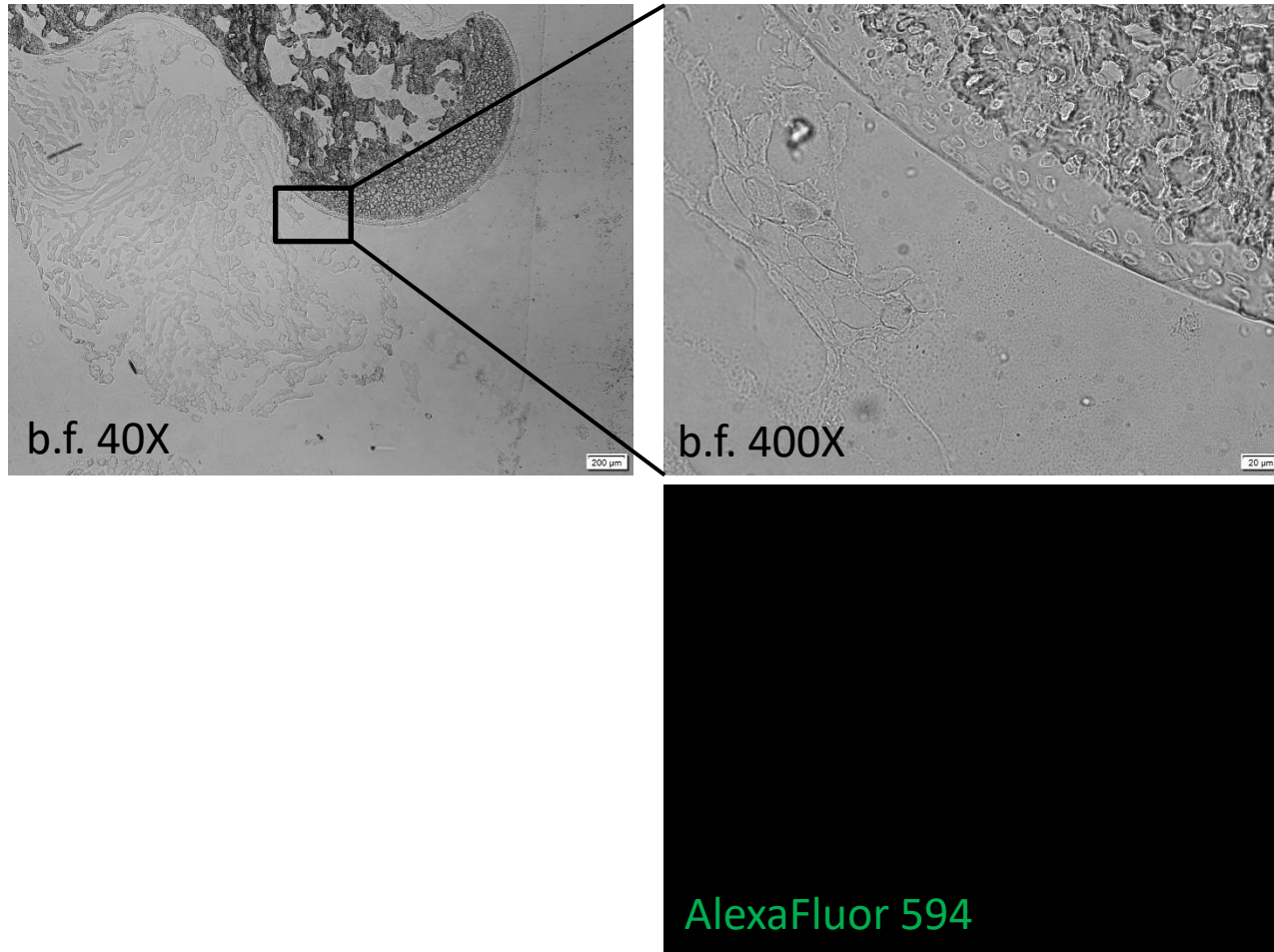

Supplement: Supplementary file 5 — Additional file 5: Figure S5. Controls for immunohistochemical staining. C57BL/6 mice (n=1) with serum induced arthritis were injected intravenously with 6 nmol LS301 at day 4 post disease induction. 6h after LS301 injection, whole body near-infrared fluorescence images were taken on the Pearl animal imaging system with λ= 820 nm, and subsequently paws and ankles were harvested and frozen for sectioning. Sections were incubated with AlexaFluor 594-conjugated secondary antibody only and viewed for fluorescence by microscopy under the Texas Red channel (Ex/Em 562±20nm/624±20nm). [file 13075_2021_2643_MOESM5_ESM.pdf]

Figure S8 | Biodistribution of LS301 shown on different scales.

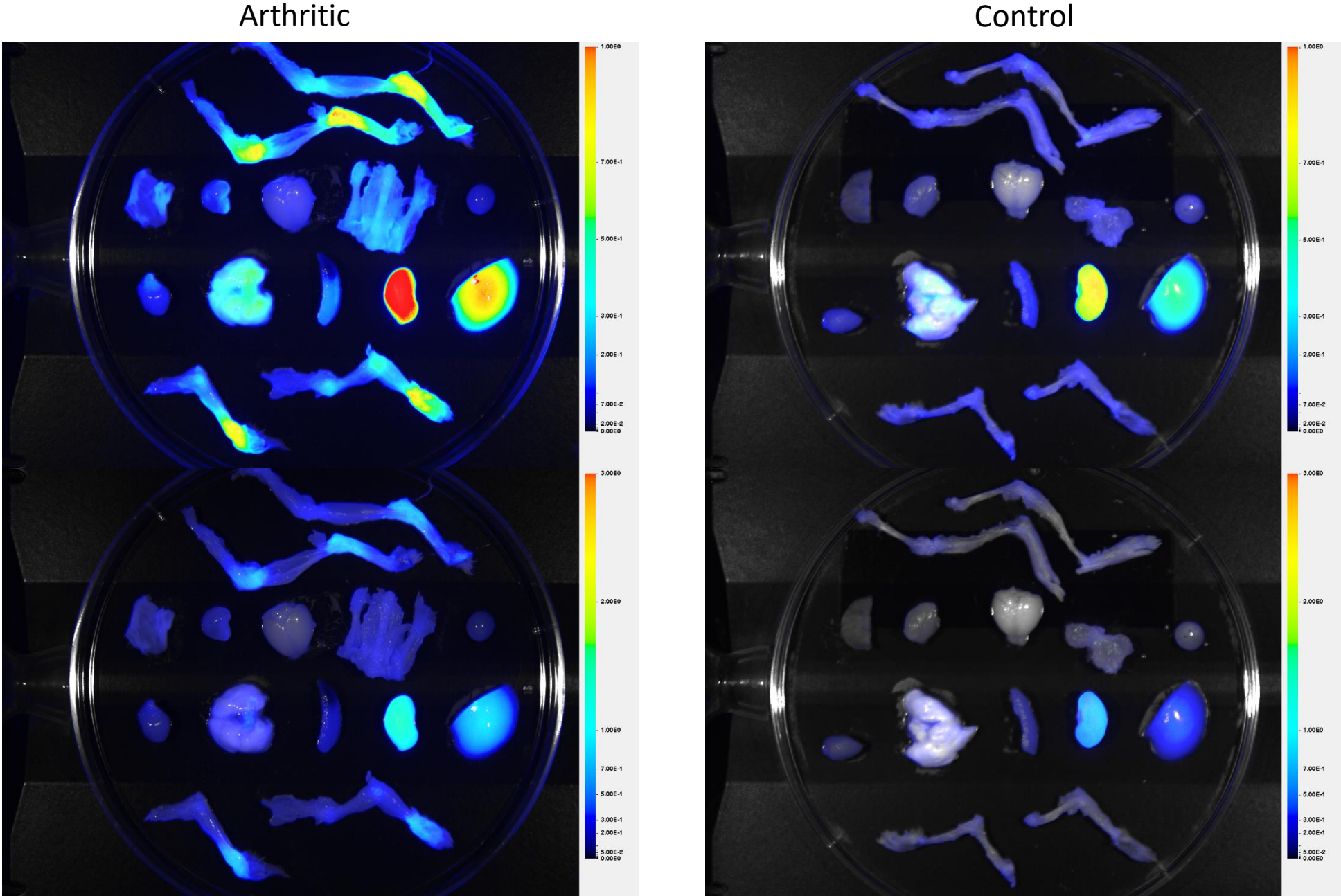

Supplement: Supplementary file 8 — Additional file 8: Figure S8. Biodistribution of LS301 shown on different scales. Shown is an example LS301 organ biodistribution from C57BL/6 mice with STA or control mice injected intravenously with LS301 (n=4 per group), shown at two different scales. Fluorescence images shown were acquired using the Pearl Small Animal Imager. Left: Arthritic mouse LS301 organ biodistribution; right: control mouse LS301 organ biodistribution. [file 13075_2021_2643_MOESM8_ESM.pdf]
