# Supplementary material for: Non-invasive monitoring of arthritis treatment response via targeting of tyrosine-phosphorylated annexin A2 in chondrocytes
Source: Arthritis Res Ther. 2021 Oct 25;23:265. doi: 10.1186/s13075-021-02643-3 (PMC8543875; doi:10.1186/s13075-021-02643-3)

Figure S3 | Regions of interest (ROIs) used for quantitation of fluorescence in individual arthritic limbs.

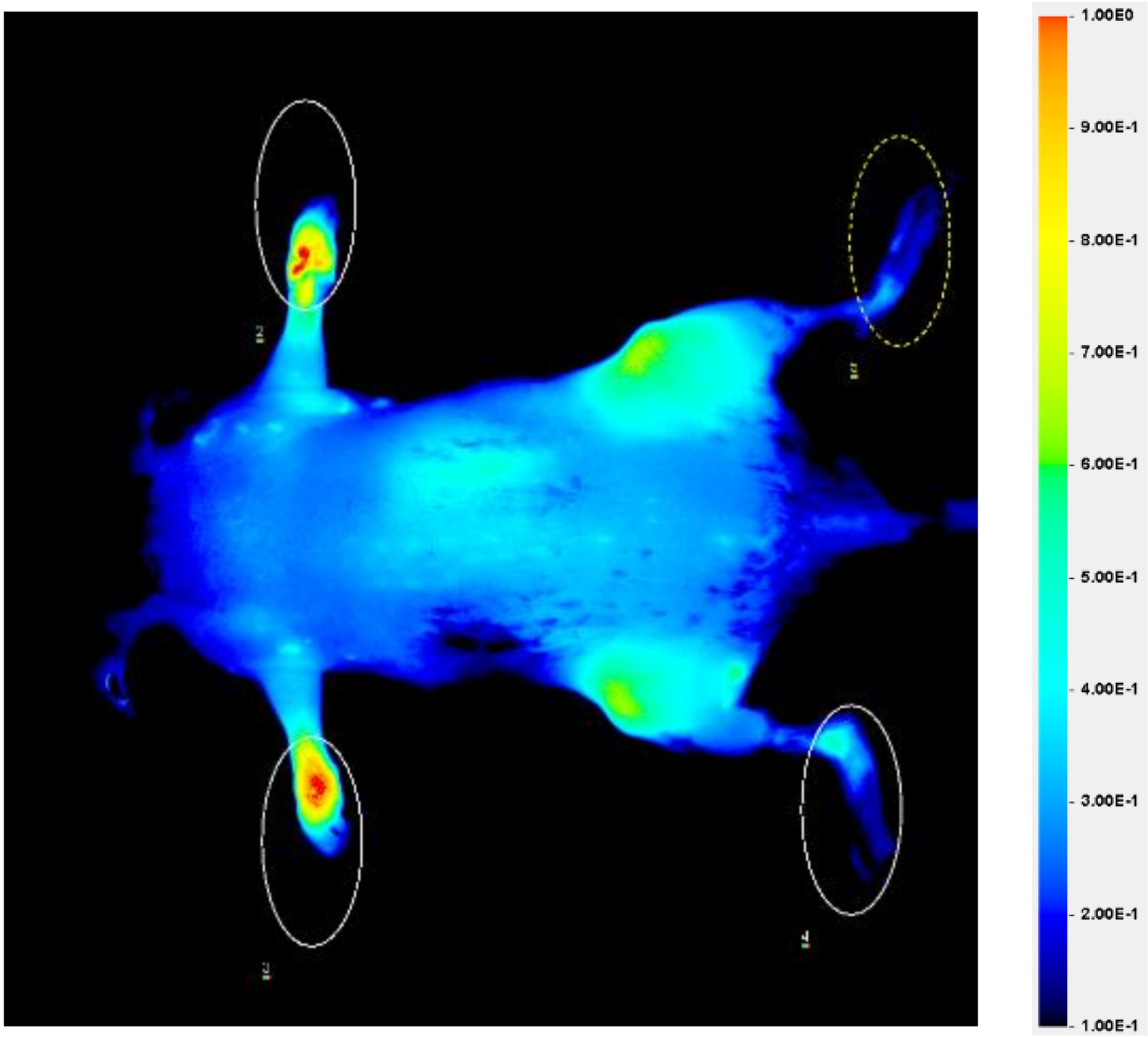

Supplement: Supplementary file 3 — Additional file 3: Figure S3. Regions of interest (ROIs) used for quantitation of fluorescence in individual arthritic limbs. Representative example of regions of interest (ROI) encompassing mouse upper extremities (all structures distal to and including the wrist) and lower extremities (all structures distal to and including the ankle) that were quantitated for LS301 fluorescence using the Pearl animal imaging system software. [file 13075_2021_2643_MOESM3_ESM.pdf]
