# Supplementary material for: Non-invasive monitoring of arthritis treatment response via targeting of tyrosine-phosphorylated annexin A2 in chondrocytes
Source: Arthritis Res Ther. 2021 Oct 25;23:265. doi: 10.1186/s13075-021-02643-3 (PMC8543875; doi:10.1186/s13075-021-02643-3)
Supplement: Supplementary file 6 — Additional file 6: Figure S6. Tissue distribution of LS301 in extraarticular regions of the mouse extremity. C57BL/6 mice with serum transfer arthritis were injected intravenously with 6 nmol LS301 at day 4 post disease induction. 6h after LS301 injection, paws and ankles were harvested and frozen for sectioning. Sections were stained with H&E and viewed for LS301 fluorescence by microscopy under the cypate channel (Ex/Em 775±25nm/845±28nm) (red). Shown are representative H&E and fluorescence images from the indicated tissues in mouse ankle. Images are representative of at least two independent experiments. (A) Skin/dermis. (B) Connective tissue. (C) Muscle. (D) Bone. (E) Bone marrow. [file 13075_2021_2643_MOESM6_ESM.pdf]

Figure S6 | Tissue distribution of LS301 in extraarticular regions of the mouse extremity.

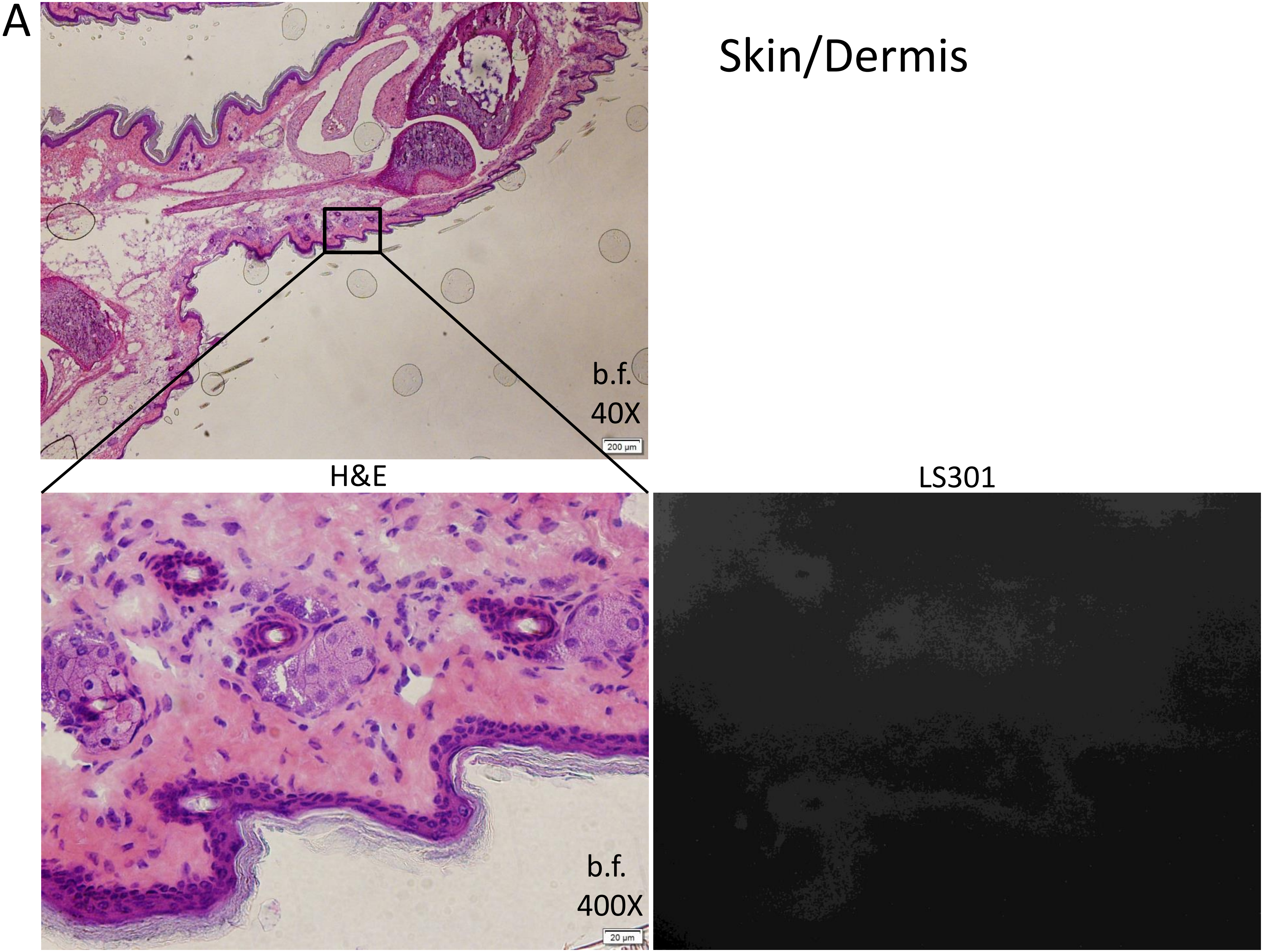
A
Skin/Dermis
b.f.
40X
200 µm
H&E
LS301
b.f.
400X
20 µm

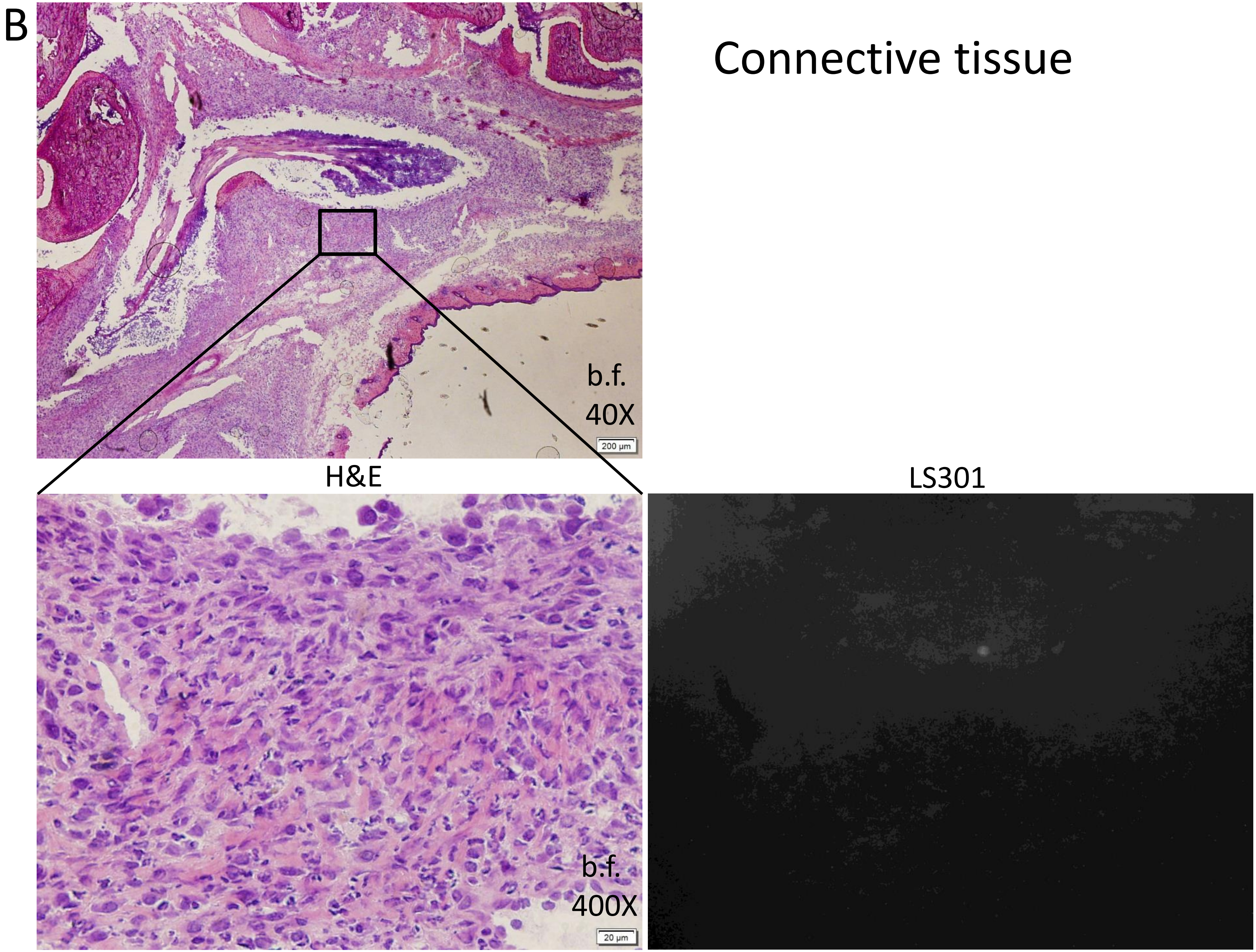
B
Connective tissue
b.f.
40X
200 μm
H&E
LS301
b.f.
400X
20 μm

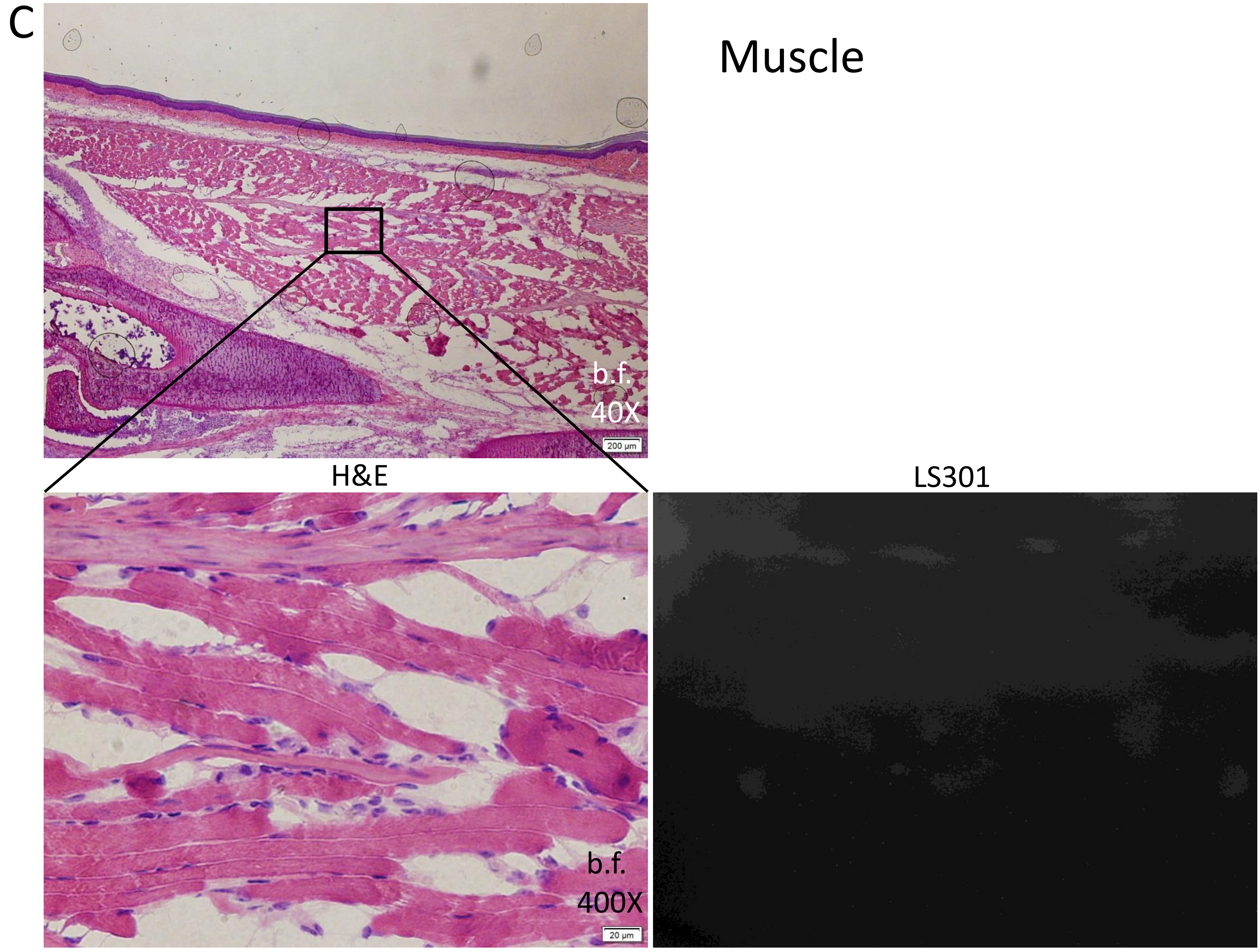

C
Muscle
b.f.
40X
200 μm
H&E
LS301
b.f.
400X
20 μm

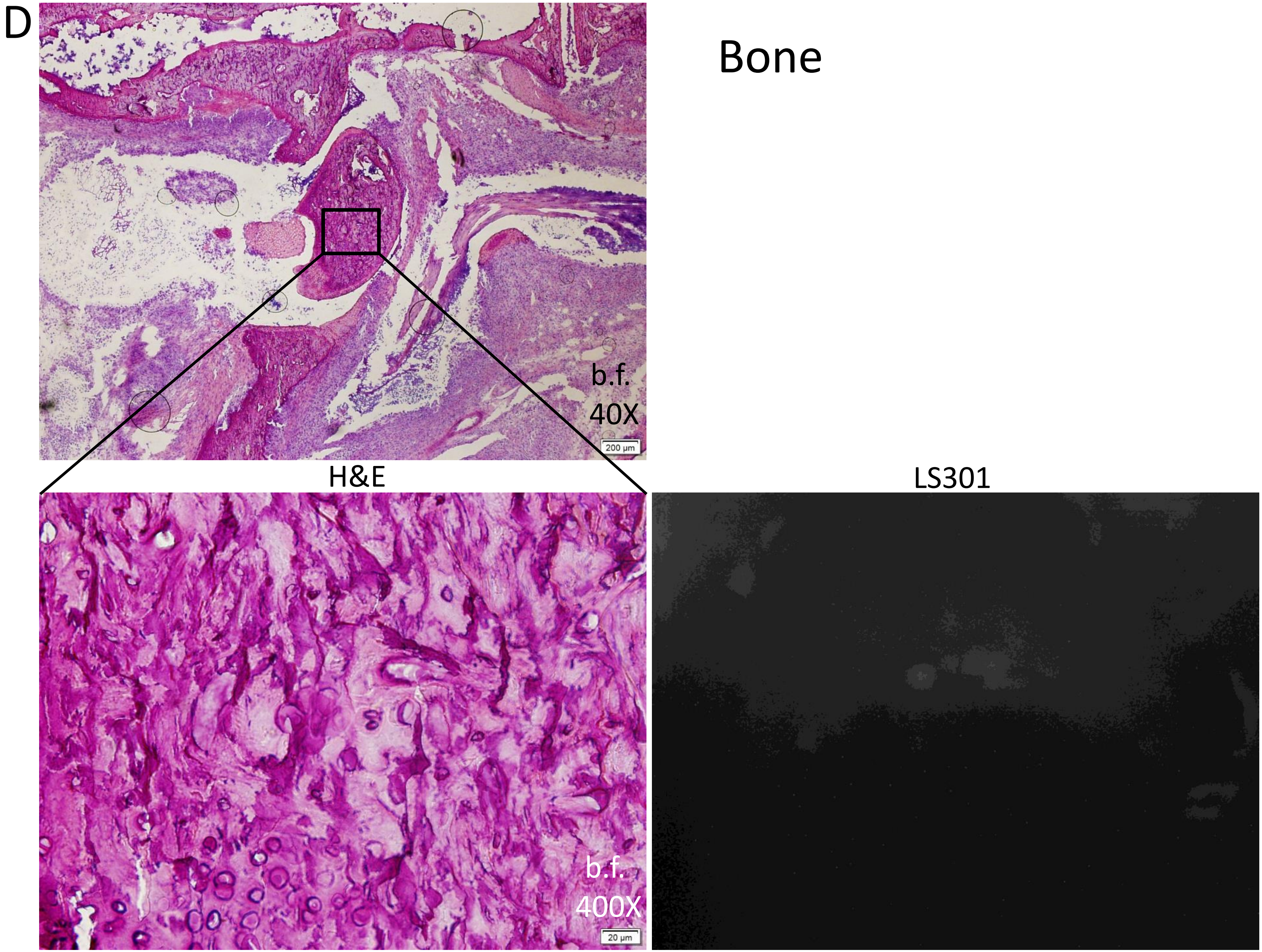
D
Bone
b.f.
40X
200 µm
H&E
LS301
b.f.
400X
20 µm

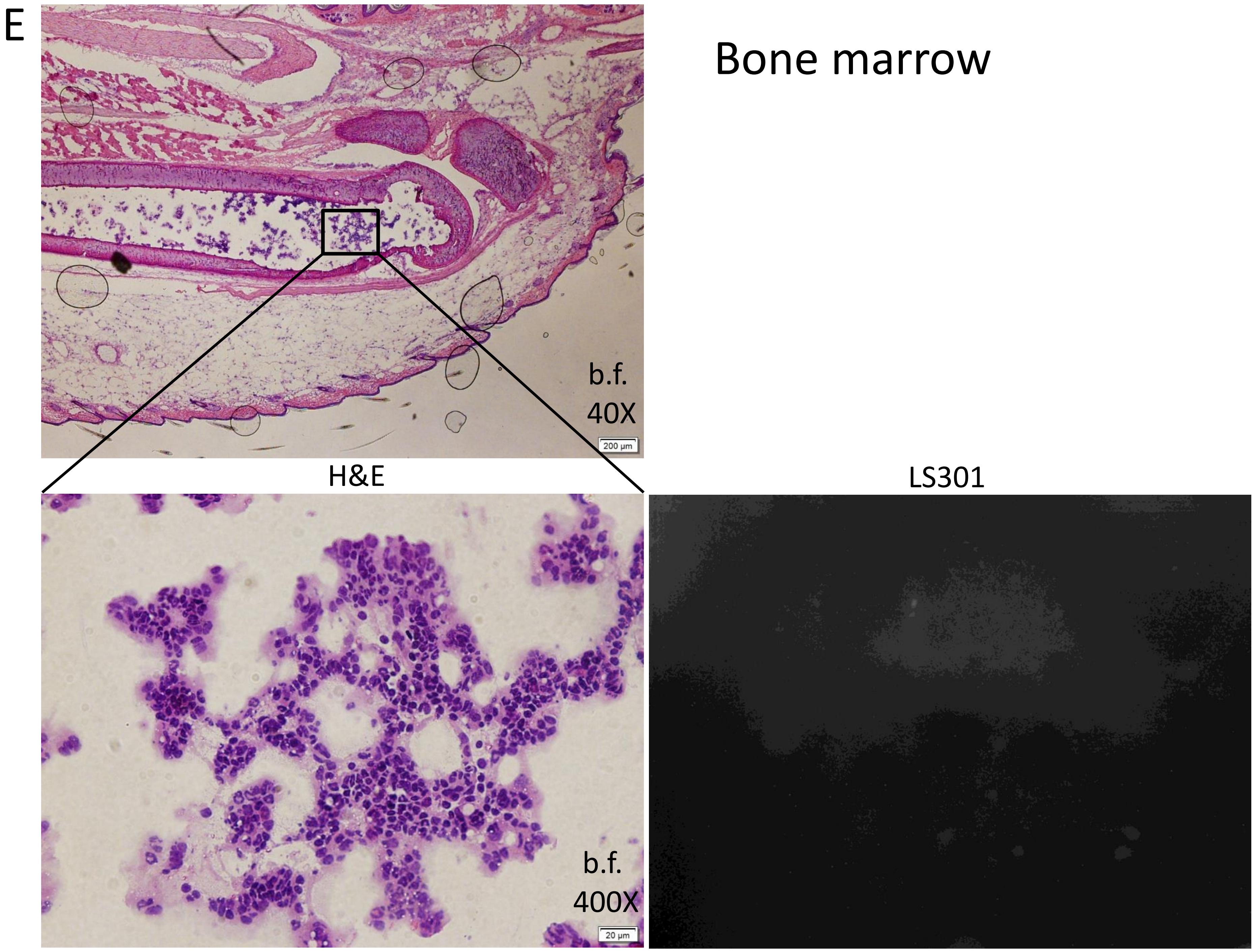
E
Bone marrow
b.f.
40X
200 µm
H&E
LS301
b.f.
400X
20 µm
