# Supplementary material for: Non-invasive monitoring of arthritis treatment response via targeting of tyrosine-phosphorylated annexin A2 in chondrocytes
Source: Arthritis Res Ther. 2021 Oct 25;23:265. doi: 10.1186/s13075-021-02643-3 (PMC8543875; doi:10.1186/s13075-021-02643-3)

Figure S7 | Relative expression levels of pANXA2 and ANXA2 in ankle and foot tissues of arthritic mice.

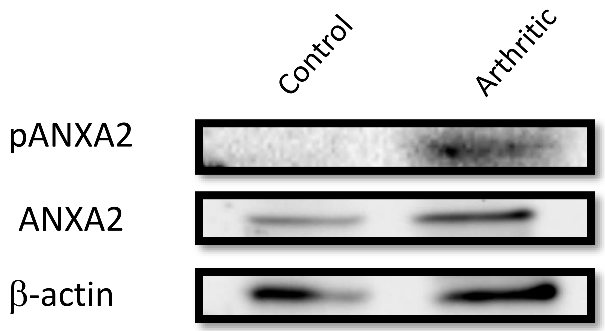

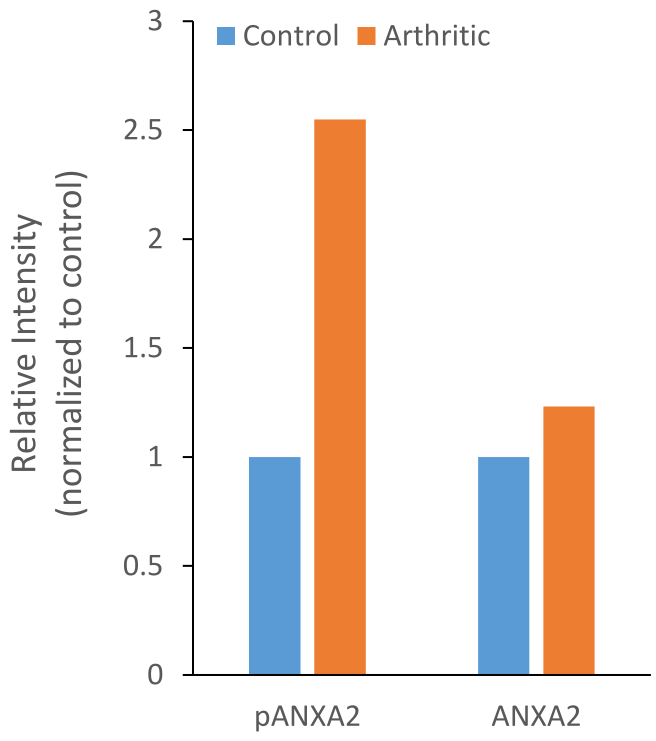

Supplement: Supplementary file 7 — Additional file 7: Figure S7. Relative expression levels of pANXA2 and ANXA2 in ankle and foot tissues of arthritic mice. C57BL/6 mice with serum transfer arthritis (n=1 per group) were sacrificed at day 8 post disease induction and ankle and foot tissue were harvested, homogenized and subjected to immunoblotting analysis for pANXA2 and ANXA2. Left: Immunoblot data for pANXA2 and ANXA2 in arthritic vs. control mouse tissue. Right: Corresponding signal quantitation using ImageJ software. [file 13075_2021_2643_MOESM7_ESM.pdf]
