# Supplementary material for: Non-invasive monitoring of arthritis treatment response via targeting of tyrosine-phosphorylated annexin A2 in chondrocytes
Source: Arthritis Res Ther. 2021 Oct 25;23:265. doi: 10.1186/s13075-021-02643-3 (PMC8543875; doi:10.1186/s13075-021-02643-3)

Figure S9 | Representative image depicting organ ROI delineation on the Pearl Small Animal Imager software.

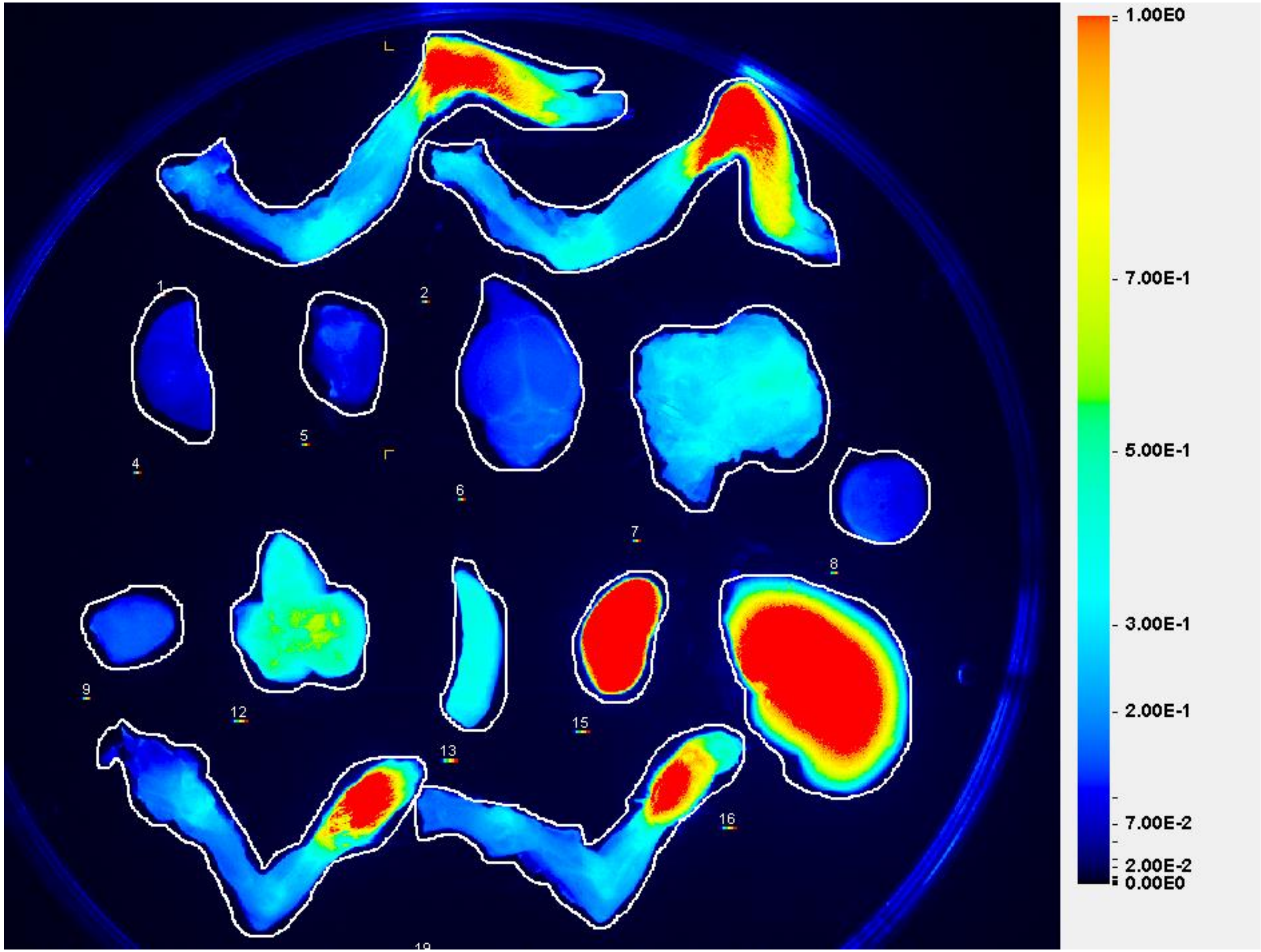

Supplement: Supplementary file 9 — Additional file 9: Figure S9. Representative image depicting organ ROI delineation on the Pearl Small Animal Imager software. Shown is an example LS301 organ biodistribution from C57BL/6 mice with STA injected intravenously with LS301 (n=4). ROIs were drawn using the freehand shape tool on the Pearl Small Animal Imager. [file 13075_2021_2643_MOESM9_ESM.pdf]
